# Evaluation of the effects of metformin on gut functions and microbiota and their contribution to improving glucose tolerance in diabetic mice

**DOI:** 10.1016/j.molmet.2025.102263

**Published:** 2025-09-29

**Authors:** Murielle Godet, Emmanuelle Meugnier, Oriane Vitalis, Nadia Bendridi, Aurélie Vieille-Marchiset, Nathalie Vega, Bérengère Benoit, Claudie Pinteur, Dominique Rainteau, David Cheillan, Marie-Caroline Michalski, Karim Chikh, Hubert Vidal

**Affiliations:** 1 Université Claude Bernard Lyon1, Laboratoire CarMeN, INSERM U1060, INRAE U1397, Fr-69310 Pierre Bénite, France; 2 Metanutribiota platform, Service de Biochimie et Biologie Moléculaire, Hôpital Lyon Sud, Hospices Civils de Lyon, Pierre-Bénite 69310, France; 3 Sorbonne Université, Centre de Recherche Saint-Antoine, INSERM U938, 75012 Paris, France; 4 Service de Biochimie et Biologie Moléculaire, Centre de Biologie et de Pathologie Est, Hospices Civils de Lyon, 69500 Bron, France

**Keywords:** Gut microbiota, Bile acids, Ceramides, FXR, Intestinal barrier, Type 2 diabetes

## Abstract

**Objectives:**

Although the mechanism of action of the antidiabetic drug metformin is still a matter of discussions, increasing evidence points to a pivotal role of the gut. Aiming to clarify whether metformin-induced changes in the intestinal tract directly contribute to metabolic improvement, we evaluated the effects of escalating doses (from 50 to 200 mg/kg/day) of metformin orally administered for 4 weeks in mice made glucose intolerant by ten weeks of high fat high sucrose diet.

**Methods:**

Several intestinal parameters were studied, including caecal microbiota composition and bile acids profile, ileal FXR signaling, abundance of GLP1-producing cells and goblet cells and blood metabolome.

**Results:**

Metformin restored glucose tolerance, fasting insulinemia and HOMA-IR index in a dose-dependent manner. Only a subset of gut-related effects, including mucus production and GLP-1 expression, exhibited a parallel dose–response relationship, suggesting a possible contribution to the observed metabolic improvements. In contrast, other changes, including ileal Fxr-Fgf15 inhibition and hepatic ceramide reduction did not scale with dose, suggesting they are not the main drivers of metformin dose-dependent effects on glycemic control. We also pointed out marked differential sensitivity of gut bacteria to metformin supporting complex interactions of the drug with the microbial ecosystem.

**Conclusion:**

Finally, metformin enhanced the proliferation of intestinal epithelium, resulting in increased length of ileal villi. Altogether, this study offers new insights into the metformin mechanism of action and revealed potential novel microbial biomarkers and targets for enhancing its therapeutic efficacy.

## Highlights

• Oral administration of metformin restored glucose tolerance and fasting insulinemia in a dose-dependent manner in glucose intolerant mice.
• Similar dose–response effect was observed for metformin-induced mucus production, endocrine differentiation and GLP-1 expression in the colon, and for the abundance of several bacterial taxa in the caecum.
• In contrast, ileal Fxr-Fgf15 inhibition and hepatic ceramide reduction did not scale with dose, suggesting they are not the main drivers of metformin dose-dependent effects on glycemic control.
• The sensitivity to metformin of gut bacteria was higly variable supporting complex interactions of the drug with the microbial ecosystem.

## 1 Introduction

Metformin (1,1-dimethylbiguanide hydrochloride) is the main oral medication and first-line therapy for type 2 diabetes mellitus (T2DM). While it is widely accepted that metformin lowers blood glucose mainly by reducing endogenous glucose production in diabetic patients, the exact mechanisms remain debated [[1], [2], [3]]. Several studies suggest a critical role of the intestinal tract, particularly the gut microbiota [[4], [5], [6], [7]]. Metformin modulates gut microbiota composition [5,7], and has been associated with increased abundance of some species such as *Akkermansia muciniphila* [8,9] which have been linked to improved barrier and metabolic function [10].

In addition to, or because of its impact on the microbiota, metformin induces several changes in intestinal functions. These include effects on glucose handling such as the inhibition of glucose absorption in the proximal small intestine [11,12], reduced gastric emptying after an oral glucose load [13,14] and stimulation of glucose uptake from the circulation and its excretion into the gut lumen [15,16]. These changes could contribute to improved glycemic control. In parallel, metformin inhibits bile acid absorption by intestinal epithelium [17,18] and modifies the bile acid pool, in part through an interplay with the change in the gut microbiota [19]. Intestinal glucose retention and increased luminal bile acid concentration are thought to stimulate the production of the incretin hormone glucagon-like peptide 1 (GLP-1) [19,20]. Furthermore, inhibition of bile acid absorption, together with modification of the bile acid profile could also lead to inhibition of the intestinal nuclear receptor FXR (Farnesoid X Receptor) [6,9] which could impact whole-body metabolic control [21]. Finally, it has been also evidenced that metformin activates goblet cells for mucin production [8], contributing to the intestinal barrier protection. While these different effects may contribute to metformin's antidiabetic action, their direct involvement in improved glucose metabolism remains uncertain.

In a recent study using mice fed a high-fat, high-sucrose (HFS) diet for 8 days, we showed that metformin counteracts HFS-induced alterations in key nutrient absorption genes in the small intestine, increases *A. muciniphila* throughout the gut, and beneficially shifts the profile of secondary bile acids in the caecum [9]. Specifically, metformin reduced deoxycholic acid (DCA) and lithocholic acid (LCA), while increasing ursodeoxycholic acid (UDCA) and tauroursodeoxycholic acid (TUDCA), alongside a marked inhibition of ileal Fxr signaling, evidenced by reduced fibrobast growth factor 15 (Fgf15) expression [9]. These findings align with data from T2DM patients treated with metformin for 3 days, where increased TUDCA and glycoursodeoxycholic acid (GUDCA) levels in stool were also linked to FXR inhibition [6]. Additionally, beyond bile acids and Fxr, changes in microbiota composition are associated with shifts in metabolites and key hormones regulating metabolic pathways [22]. Several studies report significant metabolome changes following metformin in biofluids and tissue [23], including increased production of short-chain fatty acids (SCFAs) by gut microbiota, as observed in clinical trials [24,25].

To investigate how the effects of metformin in the gut contribute to its systemic metabolic benefits, we conducted a four weeks dose–response study in obese and glucose-intolerant mice induced by a 10-week HFS diet. We aimed to establish associations between whole-body metabolic outcomes and the changes in intestinal parameters, including microbiota composition, caecal bile acids, ileal FXR signaling, blood metabolome, and the abundance of GLP1-producing cells and goblet cells in the small intestine and colon. We found that metformin effects on a subset of these intestinal parameters showed a dose-dependent variation, highlighting differential sensitivities and helping identify key intestinal mechanisms potentially driving metformin systemic benefits.

## 2 Results

### 2.1 Metformin improves metabolic disturbances in a dose–response manner in HFS fed mice

**Figure 1:**
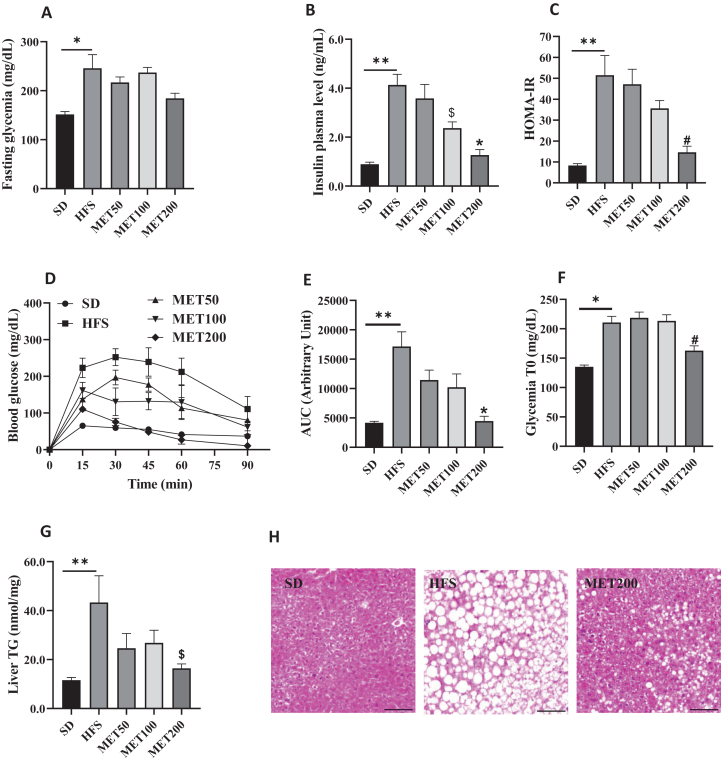
***Metformin improves metabolic disturbances in a dose-dependent manner in HFS-fed mice.* (A)** Fasting plasma glucose levels (mg/dL) measured at sacrifice after 6 h of fasting. **(B)** Fasting plasma insulin concentrations (ng/mL) measured at sacrifice after 6 h of fasting. **(C)** HOMA-IR index. **(D)** Intraperitoneal glucose tolerance test (ipGTT, 1 g of glucose/kg of body weight) performed after 6 h of fasting at week 14 of treatment. Blood glucose levels were measured every 15 min over a 90-minute period. **(E)** Area under the curve (AUC) calculated from ipGTT results and expressed in arbitrary units. **(F)** Fasting blood glucose levels (mg/dL) measured immediately before ipGTT initiation. **(G)** Hepatic triglyceride content (nmol/mg liver tissue). **(H)** Representative images of Hematoxylin/Phloxin stained liver sections: control mouse (SD), HFS-fed mouse (HFS), and HFS-fed mouse treated with 200 mg/kg of metformin (MET200). Magnification: 10 × ; scale bar: 100 μm. Data are presented as means ± SEM. ∗p < 0.05; ∗∗p < 0.01 (ANOVA) and ^$^ p < 0.05; ^#^p < 0.01 (Tukey test, MET vs HFS condition).

HFS feeding induced metabolic disturbances in adult C57BL/6J male mice, evidenced by increased fasting glucose and insulin levels (Figure 1A,B) and impaired insulin sensitivity based on HOMA-IR (Figure 1C), compared to standard chow diet (SD). Four weeks of metformin treatment tended to lower fasting glucose (Figure.1A) and almost fully corrected hyperinsulinemia in a dose-dependent manner (Figure 1B), restoring HOMA-IR at the highest dose (Figure 1C). Glucose tolerance during ipGTT (intraperitoneal glucose tolerance test), strongly impaired by HFS diet, was also corrected dose-dependently by metformin (Figure 1D,E).

Regarding body composition, metformin slightly reduced fat mass and increased lean mass, with significant effects mainly at the highest dose (Supplementary Fig. 1A and B). Liver weight was reduced in HFS-fed mice treated with metformin (Supplementary Fig. 1C), alongside a marked decrease in hepatic steatosis, as shown by triglyceride assay and lipid droplet staining (Figure 1G,H). Notably, this liver fat reduction was already evident at the lowest dose (Figure 1G). Finally, metformin dose-dependently restored caecum weight (Supplementary Fig. 1D), a change often associated with altered gut microbiota.

### 2.2 Metformin modifies caecal microbiota composition in a dose-dependent manner in HFS-fed mice

The Shannon index of alpha diversity (Supplementary Fig. 2A) was not significantly different between groups, indicating that bacterial richness was not markedly affected, although diversity tended to decrease at the highest metformin dose (MET200). However, metformin exerted a dose-dependent impact on microbiota composition at the phylum level (Supplementary Fig. 3A and B). HFS feeding increased *Bacillota* (formerly *Firmicutes*) and decreased *Verrucomicrobiota*, whereas metformin reversed these changes, significantly reducing *Bacillota* and increasing *Verrucomicrobiota*. *Bacteroidota* were globally unaffected (Supplementary Fig. 3B). Metformin also altered less abundant phyla. *Thermodesulfobacteriota*, increased by HFS diet, were nearly eliminated by metformin at the highest dose and *Pseudomonadota*, reduced by HFS, were restored in a dose-dependent manner by metformin (Supplementary Fig. 3B).

Beta diversity analysis (Supplementary Fig. 2B) confirmed distinct microbiota compositions between groups, with major effects of the HFS diet versus SD, but also clear differences between untreated and metformin-treated mice, especially at MET200. Using MaAsLin2, we identified 86 ASVs significantly different between metformin and HFS groups (with corrected q values < 0.05). These ASVs were further annotated using Nucleotide BLAST®, enabling genus and, in some cases, species-level identification (Supplementary Table 1).

**Figure 2:**
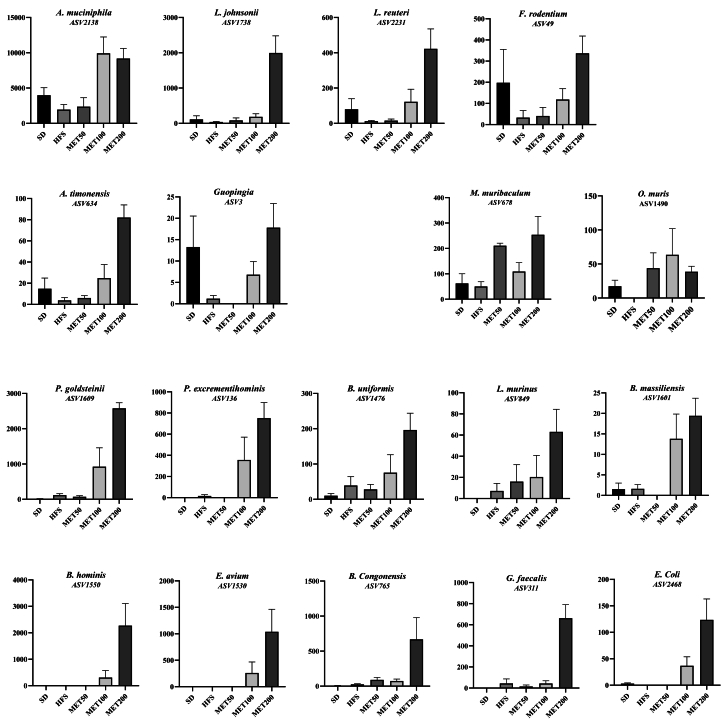
**Abundances of metformin up-regulated bacteria in the caecum, expressed as rarefied counts**. Data are presented as means ± SEM.

Metformin increased various members of the *Bacteroidaceae* (*B. uniformis*, *B. congonensis*), *Lactobacillaceae* (*L. johnsonii*, *L. reuteri*, *L. murinus*), *Lachnospiraceae* (*B. hominis*, *O. muris*, *Lachnoclostridium*), *Rikenellaceae* (*Alistipes*, *Rikenella*), *Erysipelotrichaceae* (*F. rodentium*, *Holdemania*), *Christensenellaceae* (*Candidatus*, *Guopingia*), *Enterococcaceae*, *Sutterellaceae* (*P. excrementihominis*), *Enterobacteriaceae* (*E. coli*), and *Akkermansiaceae* (*A. muciniphila*) (Supplementary Table 1). Notably, as shown in Figure 2, many up-regulated bacteria were suppressed by HFS and restored by metformin—e.g., *A. muciniphila*, *L. reuteri*, *F. rodentium*, *A. timonensis*, *Guopingia*. Others, slightly increased by HFS, were boosted by metformin in a dose-dependent manner (e.g., *B. uniformis*, *L. murinus*). Some were detected only with metformin, including *B. hominis*, *E. avium*, and *E. coli* (Figure 2). Dose-dependent responses varied: most taxa were unaffected by MET50, except *M. muribaculum* and *O. muris*, which peaked at this dose, while *L. johnsonii*, *B. congonensis*, and *G. faecalis* responded only at MET200 (Figure 2). Others responded progressively between MET100 and MET200, with *A. muciniphila* already maximally increased at MET100, indicating differential bacterial sensitivity to metformin.

**Figure 3:**
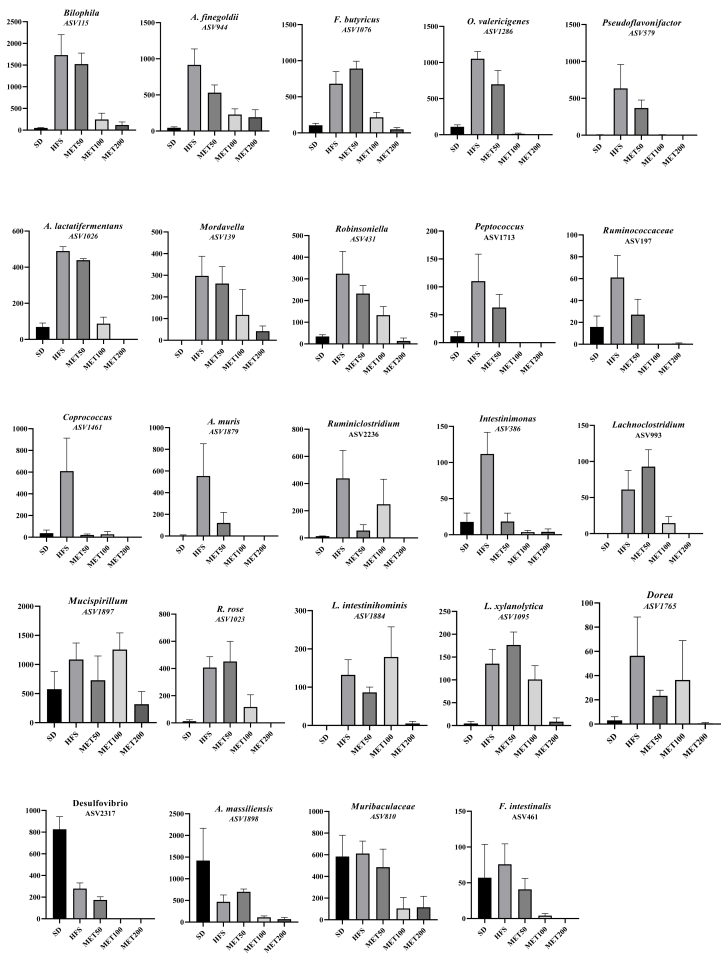
**Abundances of metformin down-regulated bacteria in the caecum, expressed as rarefied counts**. Data are presented as means ± SEM.

Conversely, metformin reduced bacteria from families such as *Lachnospiraceae*, *Oscillospiraceae*, *Rikenellaceae*, *Clostridiaceae*, *Desulfovibrionaceae*, and *Eubacteriales incertae sedis*, with *Lachnospiraceae* comprising 42 % of the significantly decreased bacteria (Supplementary Table 1). Several genera linked to metabolic disorders or type 2 diabetes (*Dorea*, *Bilophila*, *Desulfovibrio*, *Acetatifactor*, *Robinsoniella*) were down-regulated by metformin (Supplementary Table 1). Among down-regulated bacteria (Figure 3), most were increased by HFS and suppressed by metformin, returning to control or lower levels at MET200. Several taxa were highly sensitive to MET50, including *Coprococcus*, *A. muris*, *Ruminiclostridium*, *Intestinimonas*. Others, such as *A. finegoldii*, *O. valericigenes*, *Pseudoflavonifractor*, *Robinsoniella*, *Peptococcus*, showed gradual reductions from MET50 onward. *Bilophila*, *F. butyricus*, *A. lactatifermentans*, *Roseburia*, and *Dorea* were affected only at MET100 or MET200. A few taxa unaffected by HFS were reduced only at high metformin doses (e.g., *F. intestinalis*, *A. massilensis*, *Muribaculaceae*, *Desulfovibrio*) again reflecting variable sensitivity (Figure 3).

We also assessed metformin's effects in the colon and small intestine. 16S rRNA sequencing on DNA from ileal and colonic tissue revealed high concordance between caecum and colon. About 90 ASVs were regulated by MET200 in the colon (29 up-, 60 down-regulated; p < 0.05), with 61 overlapping those found in the caecum (Supplementary Fig. 4B). Key up-regulated taxa in colon included *A. muciniphila*, *L. johnsonii*, *L. reuteri*, *B. hominis*, *P. goldsteinii*, and *E. coli* whereas down-regulated taxa were mostly from *Lachnospiraceae*, *Oscillospiraceae*, *Rikenellaceae*, and *Desulfovibrionaceae* (*Bilophila*, *Desulfovibrio*) (Supplementary Fig. 4A). In the small intestine (ileum), low abundance limited the sequencing quality and statistical power. A few number of taxa showed modest metformin responses, but *A. muciniphila*, *L. johnsonii*, *L. reuteri*, *B. hominis*, and *P. excrementihominis* were clearly up-regulated (Supplementary Fig. 4C).

### 2.3 Metformin increases mucus barrier and endocrine L-cells in the colon of HFS-fed mice

**Figure 4:**
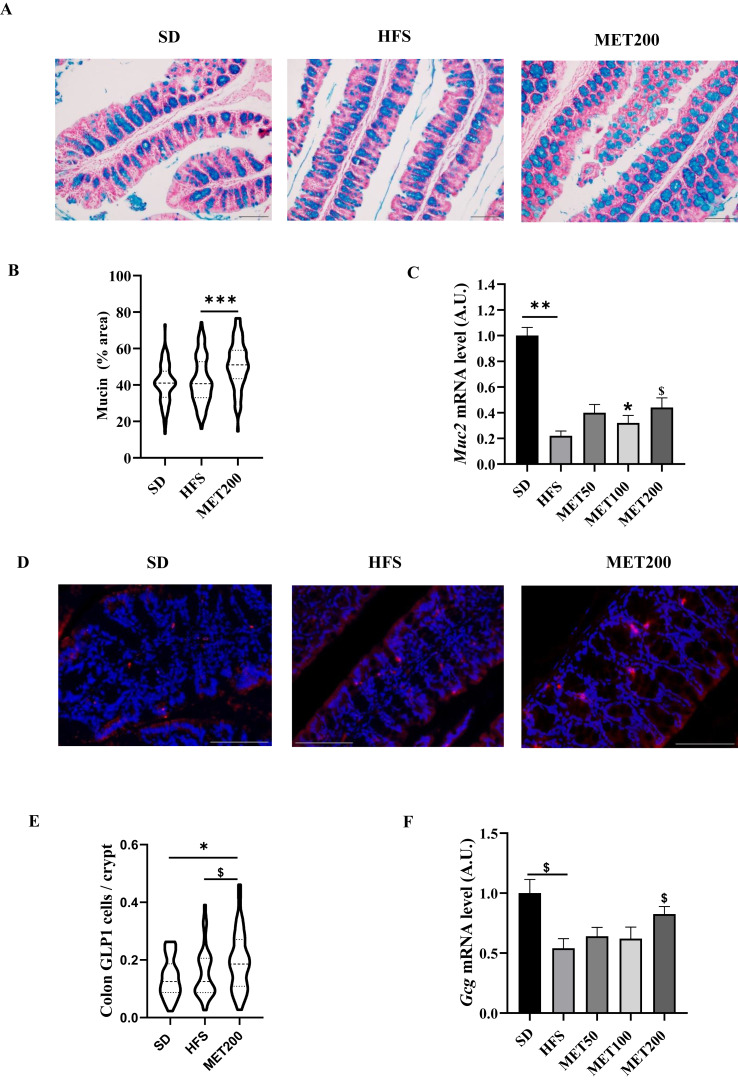
***Metformin enhances mucus barrier and increases L-cell abundance in the colon of HFS-fed mice***. **(A)** Representative images of colon sections stained with Alcian Blue, highlighting mucin-producing goblet cells. Images are shown for a control mouse (SD), an HFS-fed mouse (HFS), and an HFS-fed mouse treated with 200 mg/kg of metformin (MET200). Magnification: 20 × ; scale bar: 100 μm. **(B)** Violin plots showing the percentage of Alcian Blue-positive vesicles (mucus) area covering the epithelium. **(C)** Relative expression of *Muc2* mRNA in colon samples measured by RT-qPCR (data are means ± SEM and expressed as arbitrary units, normalized to *Tbp* expression). **(D)** Representative images of colon sections stained for GLP-1-positive L-cells (pink), with nuclei counterstained in blue using DAPI. Images are shown for each treatment group as in panel A. Magnification: 40 × ; scale bar: 100 μm. **(E)** showing the quantification of GLP-1-positive cells per colonic crypt. **(F)** Relative expression of *Gcg* mRNA in colon samples measured by RT-qPCR (means ± SEM, arbitrary units as in C). In the violin plots, horizontal lines represent the data quartiles. ∗p < 0.05; ∗∗p < 0.01; ∗∗∗p < 0.001 (ANOVA) and ^$^ p < 0.05 (Tukey test, MET vs HFS condition).

Bacteria such as *Akkermansia*, *Lactobacilli* and *Blautia* (increased by metformin) have been linked to improved epithelial barrier function and enhanced GLP-1 production by L-cells. We first assessed mucus content and goblet cells in the colon. Figure 4A shows that mucus-filled vesicles (blue-stained) were increased in metformin-treated mice. Quantitatively, although the HFS diet did not significantly affect mucus vesicle percentage, it was markedly increased after 4 weeks of MET200 treatment (Figure 4B). *Muc2* gene expression, encoding mucin 2, was significantly reduced by HFS feeding and dose-dependently restored by metformin in the colon (Figure 4C).

Regarding entero-endocrine cells in colon, immunohistology revealed that GLP-1-positive cell numbers were unchanged by HFS diet but increased with metformin treatment (Figure 4D,E). At the transcriptional level, HFS diet lowered *Gcg* expression (encoding GLP-1), while metformin restored *Gcg* expression at the highest dose (MET200) (Figure 4F).

### 2.4 High-fat sucrose diet and metformin affect intestinal epithelium morphology and differentiation in mice

**Figure 5:**
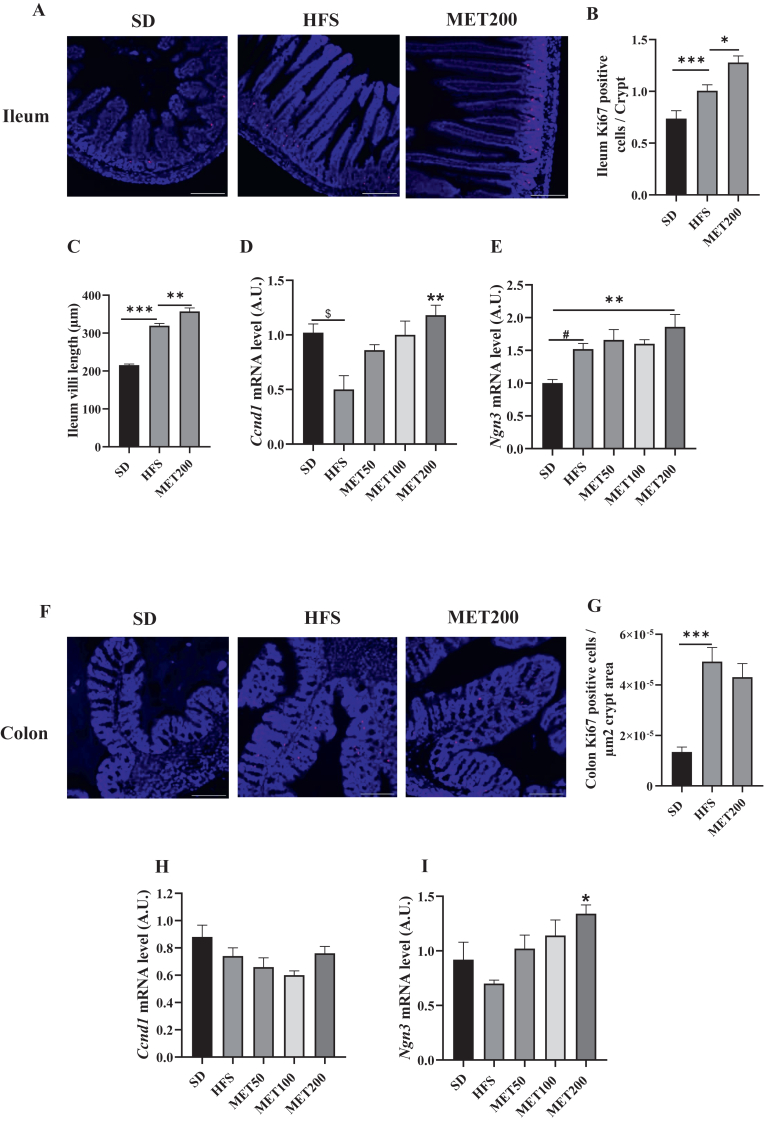
**High-fat sucrose diet and metformin alter intestinal epithelial morphology and differentiation. (A)** Representative images of ileal sections stained for Ki67-positive cells (pink), with nuclei counterstained in blue using DAPI. Images are shown for control mouse (SD), HFS-fed mouse (HFS), and HFS-fed mouse treated with 200 mg/kg of metformin (MET200). Magnification: 20 × ; scale bar: 100 μm. **(B)** Quantification of Ki67-positive cells within ileal crypts. **(C)** Villi length in ileal sections, expressed in μm. **(D**–**E)** Relative expression of *Ccnd1* (D) and *Ngn3* (E) mRNA in ileal samples, measured by RT-qPCR and normalized to *Tbp* expression. **(F)** Representative images of colonic sections stained for Ki67-positive cells (pink), with DAPI nuclear counterstaining (blue). Images are shown as in panel A. Magnification: 20 × ; scale bar: 100 μm. **(G)** Quantification of Ki67-positive cells per μm^2^ of colonic crypt area. **(H–I)** Relative expression of *Ccnd1* (H) and *Ngn3* (I) mRNA in colonic samples, measured by RT-qPCR and normalized to *Tbp* expression. ∗p < 0.05; ∗∗p < 0.01; ∗∗∗p < 0.001 (ANOVA) and #p < 0.01 (Tukey test, MET vs HFS condition).

In addition to goblet cell maturation, and mucus production, recent findings suggest that metformin protects the intestinal barrier by promoting epithelial cell proliferation [26]. We thus quantified Ki67, a classical proliferation marker. Immunohistology showed that HFS raised Ki67-positive cell numbers in ileal crypts and that metformin further amplified this effect (Figure 5A,B). Importantly, the length of the ileal villi was increased by HFS diet, an effect further enhanced by metformin (Figure 5C). We then measured mRNA levels of *Ccnd1* (cyclin D1), a key proliferation factor, and *Ngn3* (neurogenin 3), critical for enteroendocrine cell differentiation. Unlike Ki67, *Ccnd1* expression was reduced by HFS in the ileum, and metformin dose-dependently restored it (Figure 5D). *Ngn3* expression increased with HFS but was not altered further by metformin (Figure 5E).

In the colon, HFS increased Ki67-positive cells in crypts, but metformin had no additional effect (Figure 5F,G). *Ccnd1* expression was slightly reduced by HFS and unaffected by metformin (Figure 5H). In contrast, *Ngn3* expression increased dose-dependently with metformin (Figure 5I), aligning with the rise in GLP-1-positive L-cells in the colon (Figure 4D,E).

### 2.5 Metformin modifies the bile acid pool in the gut and inhibits the Fxr-Fgf15 pathway in the ileum of HFS-fed mice

**Figure 6:**
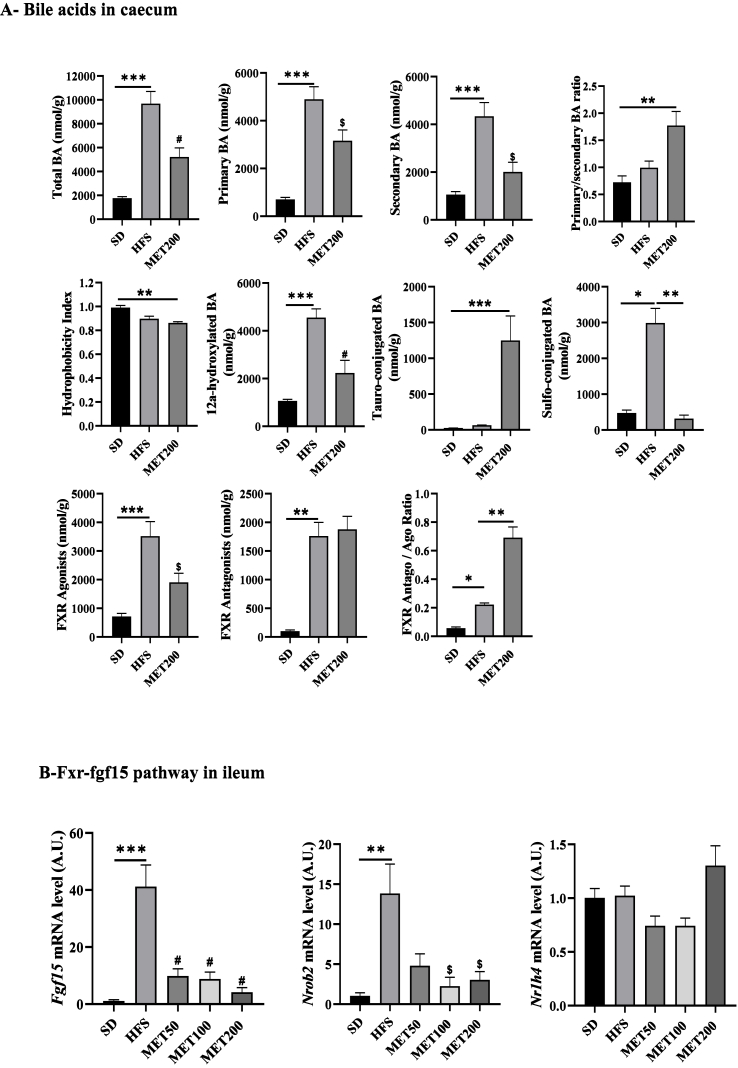
***Metformin modulates caecal bile acid pool and inhibits Fxr–Fgf15 signaling pathway in the ileum of HFS-fed mice.* (A)** Quantification of bile acids in caecal samples by HPLC-MS/MS, expressed as nmol of bile acids per gram of tissue. **(B)** Expression levels of genes involved in the Fxr–Fgf15 signaling pathway in ileal tissue. From left to right: *Fgf15*, *Nrob2*, and *Nr1h4* mRNA, measured by RT-qPCR, normalized to *Tbp* expression and expressed in arbitrary units. Data are presented as means ± SEM. ∗p < 0.05; ∗∗p < 0.01, ∗∗∗p < 0.001 (ANOVA) and ^$^ p < 0.05; ^#^p < 0.01 (Tukey test, MET vs HFS condition).

Gut microbiota is a key determinant of bile acid metabolism, driving the formation of secondary bile acids. Bile acid profiling was performed on caecal content at the end of treatment. As shown in Figure 6A, HFS diet significantly increased total bile acid levels, affecting both primary (CDCA, CA, βMCA) and secondary (DCA, LCA, HDCA, ωMCA, UDCA) species. After 4 weeks of treatment, metformin (MET200) attenuated this effect, leading to an increased primary-to-secondary bile acid ratio and reduced hydrophobicity index (Figure 6A).

The caecal abundance of 12α-hydroxylated bile acids (12αOH BA), considered deleterious, was strongly increased by HFS and reduced by metformin. Metformin also shifted the conjugation pattern, increasing tauro-conjugated and decreasing sulfo-conjugated bile acids (Figure 6A). In addition, the sum of bile acids known as FXR agonists (CDCA, CA, ωMCA, HDCA), which were elevated under HFS, were reduced by metformin, while the sum of FXR antagonists (βMCA, UDCA, TUDCA, TMCA) remained unchanged, resulting in a significantly increased antagonist/agonist ratio (Figure 6A). The individual level of bile acid species is shown in Supplementary Fig. 5. Notably, metformin strongly reduced the relative adundance of CA-7S (cholic acid-7-sulfate) and ωMCA, while TMCA, a well-known FXR antagonist, was elevated.

We next assessed the Fxr-Fgf15 pathway in the ileum. *Fgf15* and *Nrob2* (*Shp*), direct Fxr target genes, were strongly downregulated by metformin, whereas *Nr1h4* (encoding Fxr) was unchanged (Figure 6B). This molecular signature aligned with the observed bile acid shifts. Importantly, Fxr pathway inhibition was already evident at the lowest concentration of metformin and was not dependent of the dose (Figure 6B).

### 2.6 Metformin reduced ceramide concentrations in the liver of HFS-fed mice

**Figure 7:**
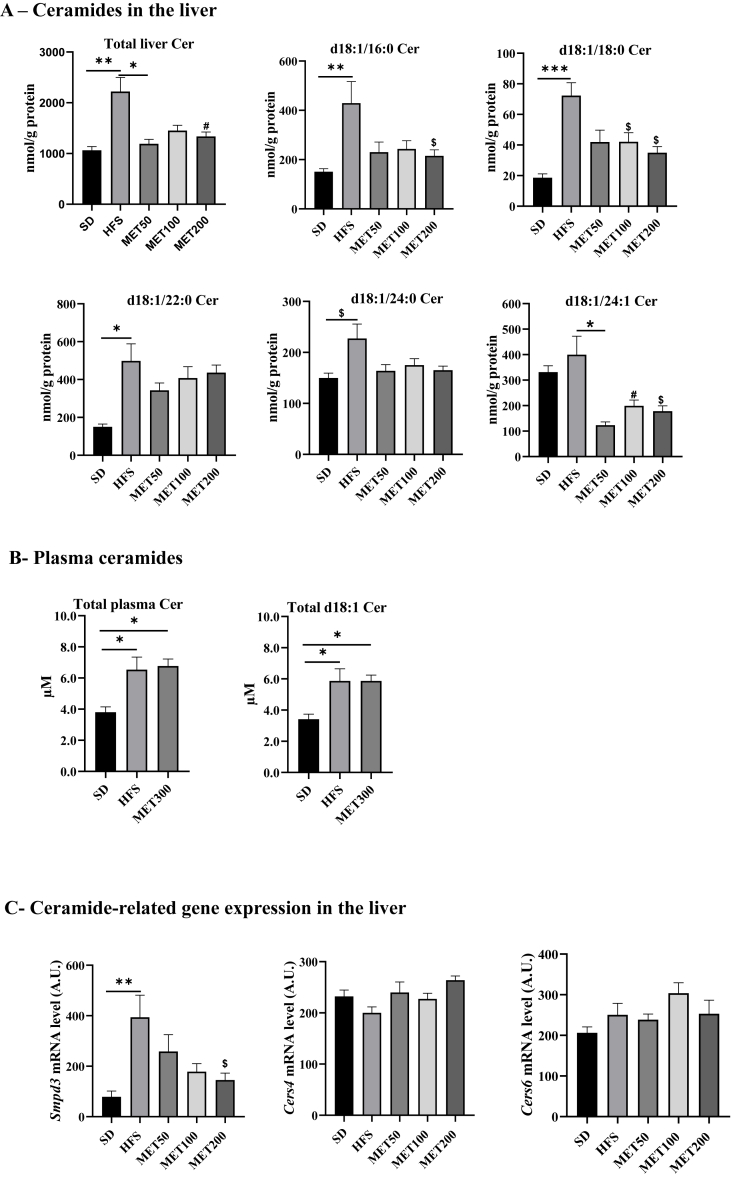
**Metformin reduces hepatic ceramide levels. (A)** Quantification of ceramide species in liver samples using LC-MS/MS, expressed as nmol of ceramides per gram of protein. **(B)** Quantification of ceramides in plasma samples using the MxP® Quant 500 Kit (Biocrates), expressed in μM. **(C)** Expression levels of genes involved in ceramide metabolism in liver tissue (*Smpd3*, *Cers4*, and *Cers6*), measured by RT-qPCR, normalized to *Tbp* expression and expressed in arbitrary units. Data are presented as means ± SEM. ∗p < 0.05; ∗∗p < 0.01, ∗∗∗p < 0.001 (ANOVA) and ^$^ p < 0.05; ^#^p < 0.01 (Tukey test, MET vs HFS condition).

Inhibition of Fxr in the ileum improves metabolic regulation and insulin resistance in rodents, possibly via reduced ceramide production and hepatic accumulation [21]. We therefore quantified liver ceramide species. As shown in Figure 4, Figure 7 a 4-week metformin treatment significantly decreased several ceramide species in HFS-fed mice, especially C16- and C18-ceramides, which are strongly linked to insulin resistance and impaired insulin signaling [27]. Longer-chain ceramides (C22, C24) were also lowered by metformin, although not significantly (Figure 7A). Additionally, C24:1 ceramide, which has been associated with inflammation and cardiometabolic risk, was not increased by HFS but was significantly reduced by metformin. Notably, these effects were not dose-dependent and were observed even at the lowest metformin dose (Figure 7A).

To assess plasma ceramides, we conducted a parallel experiment using metformin at 300 mg/kg (MET300), following the same design (10-week HFS diet + 4-week treatment). Supplementary Fig. 6 confirms that MET300 similarly affected fasting glycemia, insulinemia, and ipGTT compared to MET200 (Supplementary Fig. 6A vs Figure 1D,E). Inhibition of the Fxr-Fgf15 axis in the ileum was also replicated at this dose (Supplementary Fig. 6B vs Figure 6B). Under these conditions, plasma ceramides were quantified by LC-MS/MS. HFS increased total and various d18:1 ceramide species, but unlike in the liver, plasma ceramide levels were not significantly altered by metformin (Figure 7B), suggesting a liver-specific action of metformin on ceramide metabolism. Supporting this, *Smpd3* expression (encoding sphingomyelin phosphodiesterase 3) in the liver was upregulated by HFS and dose-dependently inhibited by metformin (Figure 7C), while *Cers4* and *Cers6* expression remained unchanged (Figure 7C).

### 2.7 Metformin modifies plasma concentrations of microbiota-derived metabolites in HFS-fed mice

**Figure 8:**
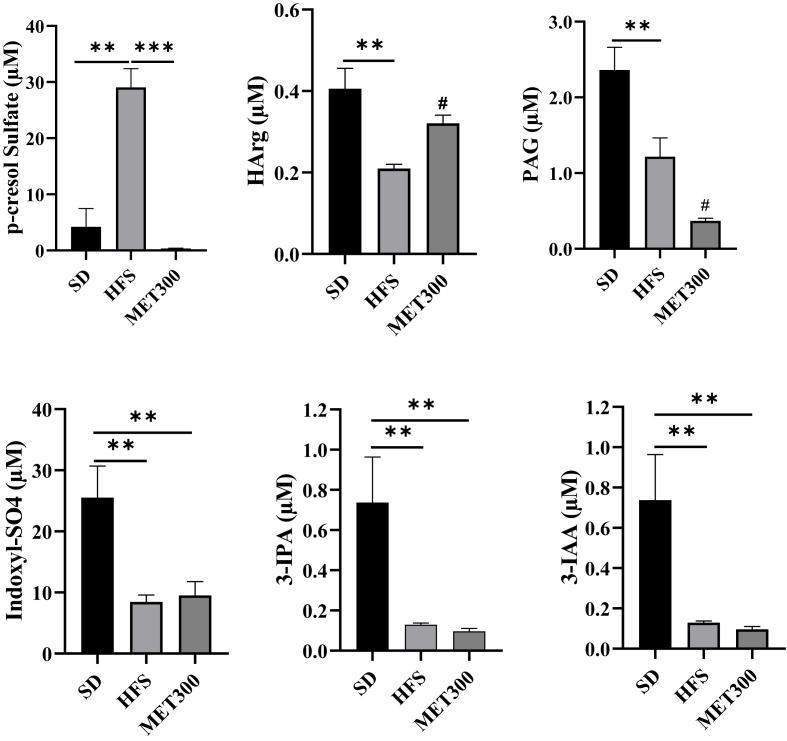
***Metformin alters the plasma concentration of selected microbiota-derived metabolites in HFS-fed mice***. Quantification of plasma microbiota-derived metabolites was performed using the MxP® Quant 500 Kit (Biocrates) and results are expressed in μM. From left to right and top to bottom: *p*-cresol sulfate, homo-l-arginine (HArg), phenylacetylglycine (PAG), indoxyl sulfate (Indoxyl-SO_4_), indole-3-propionic acid (3-IPA), and indole-3-acetic acid (3-IAA). Data are presented as means ± SEM. ∗∗p < 0.01, ∗∗∗p < 0.001 (ANOVA); ^#^p < 0.01 (Tukey test, MET vs HFS condition).

Further plasma metabolome analyses in MET300-treated mice revealed that metformin altered concentrations of several metabolites compared to HFS controls. Using PLS-DA (Supplementary Fig. 7A), we identified a metabolite signature distinguishing the HFS and MET300 groups. Key discriminant metabolites included *p*-cresol sulfate, homoarginine, and various triglyceride species with at least 4 unsaturations (Supplementary Fig. 7B). The *p*-cresol sulfate, markedly increased by HFS feeding, was completely normalized by metformin (Figure 8). Metformin also modulated homoarginine (HArg) and phenylacetylglycine (PAG) levels. Conversely, several indole metabolites (indoxyl-SO4, 3-IPA and 3-IAA, known as microbial byproducts of tryptophan) were strongly reduced under HFS diet but not restored by metformin treatment (Figure 8).

## 3 Discussion

The mechanism of action of the antidiabetic drug metformin remains under discussion, although increasing evidence points to a pivotal role of the gut [[3], [4], [5], [6], [7], [8], [9]]. To clarify how metformin-induced changes in the intestinal tract contribute to glucose metabolism improvement, we evaluated the effects of escalating doses of metformin administered for 4 weeks in mice with metabolic impairments induced by 10 weeks of HFS diet. Metformin restored glucose tolerance, fasting insulinemia and HOMA-IR in a dose-dependent manner. Fasting glycemia was only slightly reduced at the highest dose. We aimed to determine whether several metformin-driven gut parameters followed a similar dose–response trend.

The intestinal FXR pathway has recently gained attention for its role in metabolic regulation. Intestine-specific *Fxr* gene invalidation [28] and selective intestinal FXR inhibitors such as caffeic acid phenethyl ester or glycine-βMCA protect against diet-induced metabolic alterations in rodents [21,29]. We recently showed that a probiotic mixture improving glucose metabolism in HFS-fed mice was associated with ileal FXR inhibition [30]. A role for intestinal FXR in metformin's mechanism is supported by studies in type 2 diabetic patients, where 3-day metformin treatment increased fecal levels of glycoursodeoxycholic acid (GUDCA), an FXR antagonist [6]. Inhibition of Fxr in the ileum downregulates its target genes, notably *Fgf15* (the rodent ortholog of human *FGF19*), a key regulator of bile acid, lipid, and glucose homeostasis [31]. We previously reported that metformin markedly suppresses *Fgf15* expression in HFS-fed mice after 8 days [9], a finding consistent with decreased Fgf15 in metformin-treated diabetic rats [32] and reduced plasma FGF19 in patients after short-term metformin treatment [6].

In the present study, metformin robustly inhibited the Fxr–Fgf15 axis in HFS-fed mice, but independently of the concentration used since maximal inhibition occurred already at the lowest dose (MET50). This effect may be the consequence of an inhibition of the ileal transport of bile acids by metformin as classically reported both in animal and human studies [[17], [18], [19], [20]]. However, in mice fed an HFS diet, we found a slight reduction of the total amount of caecal bile acids in the presence of metformin, together with a marked increase in the tauro-conjugated forms and changes in bile acid profile favoring FXR antagonists over agonists, compared to the HFS condition (Figure 6A and Supplementary Fig. 5). These observations suggest that inhibition of the Fxr–Fgf15 axis in the ileum of metformin-treated HFS mice may result, at least in part, from the modification of luminal bile acid composition. However, since we did not assess the dose–response changes in bile acid profile, a direct link cannot be confirmed. Alternatively, metformin may directly inhibit Fxr via AMPK activation, as previously shown in liver and ileum [33]. Whatever the mechanism, the well-known strong intestinal retention of metformin [34] could potentially explained why Fxr inhibition was maximal even at the lowest dose.

Inhibition of intestinal Fxr has been linked to reduced ceramide synthesis, a pathway strongly associated to insulin sensitivity [21,29]. Xie et al. [21] showed that intestinal Fxr blockade reduced ceramide production in gut and liver. In our study, metformin lowered hepatic ceramide concentrations, particularly C16:0, C18:0, and C24:1 species, which have been associated with insulin resistance in human liver [35]. Like for ileal Fxr inhibition, this effect was dose-independent, supporting the proposed link between intestinal Fxr and the ceramide pathway [21]. Interestingly, plasma ceramides remained unchanged upon metformin, suggesting a liver-specific effect. Supporting this, *Smpd3*, encoding the key enzyme converting sphingomyelin to ceramide, was significantly downregulated by metformin in liver tissue. Unlike ceramide levels, the effect on *Smpd3* expression was dose-dependent, suggesting additional regulatory mechanisms. Together, these findings support a model in which metformin inhibits the intestinal Fxr–Fgf15 axis, via modulation of the luminal bile acid metabolism and absorption, and/or AMPK activation, leading to reduced hepatic ceramide levels. Given the known roles of ceramides in insulin resistance [27] and *Fgf15/19* in metabolic regulation [31], these changes likely contribute to metformin anti-diabetic effects. However, their lack of dose-dependence suggests they are not the main drivers of the dose-dependent effects on glucose tolerance and fasting insulinemia.

Several studies have shown that metformin increases GLP-1 (glucagon-like peptide-1) secretion in both rodents [36,37] and humans [13,14], through entero-endocrine L-cell stimulation [36]. In our study, metformin led to increased *Gcg* expression and to a higher number of GLP-1-positive cells in the colon. This was paralleled by an induction of *Ngn3*, encoding neurogenin 3, a key transcription factor driving endocrine differentiation in the colon. Notably, *Ngn3* expression followed a clear dose–response to metformin. However, directly linking GLP-1 and metformin's metabolic effects remains challenging because GLP1 concentration was not measured in the present work and previous studies using GLP-1 receptor knockout mice showed that circulating GLP-1 levels do not fully account for metformin's impact on glucose tolerance in rodent models [37].

We also observed significant effects of metformin on intestinal morphology and barrier integrity. Metformin increased ileal villus length and the number of goblet cells in colonic crypts. Similar results were described in a mouse model of radiation-induced enteropathy [26], where metformin exerted protective effects on the intestinal barrier. Our findings suggest that metformin enhances gut epithelial renewal and defense in HFS-fed mice as well. Notably, *Ccnd1* (Cyclin D1), a regulator of epithelial proliferation, was dose-dependently induced by metformin in the ileum, supporting a role in villus elongation.

Gut microbiota is central to epithelial barrier maintenance, and metformin's protective effects in irradiation models were lost upon antibiotic-induced microbiota depletion [38], indicating that microbiota plays a critical role in metformin action, as also supported by a number of other studies [[4], [5], [6], [7], [8], [9]]. In line, we observed important metformin-induced changes in caecal microbiota composition. Most of the taxa affected by metformin in this study overlapped with those previously identified in rodents and humans [39]. In most cases, metformin reversed HFS-induced alterations. A limited number of bacteria were modified independently of the diet, including *B. hominis*, *E. avium*, and *E. coli* (increased), and *F. intestinalis* and a *Muribaculaceae* member (decreased).

In addition to a strong increase in *A. muciniphila*, metformin raised the abundance of *L. johnsonii*, *L. reuteri*, *L. murinus*, *F. rodentium*, *C. guopingia*, *B. uniformis*, *B. hominis*, and *P. goldsteinii*. These species are generally associated with gut health and contribute to beneficial metabolic effects through SCFA production, gut barrier reinforcement, hormone regulation, and inflammation control [23,39,40]. Some strains are under development as next-generation probiotics for metabolic diseases [30,[41], [42], [43], [44], [45]]. Conversely, metformin reduced many taxa from *Lachnospiraceae* and *Oscillospiraceae*. Most downregulated genera have been linked to obesity or type 2 diabetes, such as *Dorea* [46], *Bilophila* [47], *Desulfovibrio* [39], *Acetatifactor* [48], *Robinsoniella* [49], and *Lachnoclostridium* [50]. Surprisingly, some metformin-suppressed bacteria like *Roseburia* [51], *Intestinimonas* [52], and *Alistipes* [53] have been associated with metabolic benefits. This could be related to strain-specific properties or to context-dependent effects.

Metformin induced distinct dose–response patterns among gut bacteria. Some taxa, like *Coprococcus*, *Acetatifactor muris*, *Ruminiclostridium*, and *Intestinimonas* (downregulated), or *Otoolea muris* (upregulated), responded strongly to the lowest dose. Others, including *L. johnsonii*, *B. hominis*, *B. congogenis*, and *G. faecalis*, required the highest dose for modulation. Several bacteria exhibited clear dose-dependent responses, such as *L. reuteri*, *F. rodentium*, *A. timonensis*, *B. uniformis*, *P. goldsteinii*, *P. excrementihominis*, and *Guopingia* (upregulated), and *Mordavella*, *Robinsoniella*, *Bilophila*, *A. finegoldii*, and *A. lactatifermentans* (downregulated). These dose-dependent responses suggest possible link between the regulation of specific bacterial species and metformin's effects on fasting insulin levels and glucose tolerance.

Many of the upregulated bacteria are known for their beneficial roles in gut health and metabolic regulation, including SCFA and indole production, vitamin biosynthesis, and modulation of bile acids and inflammation [22,23,54]. For example, a *L. reuteri* strain encodes over 200 genes related to metabolite production, including acetate, butyrate, propionate, folate, and indoles [55]. In the present study, metabolomics analysis revealed that metformin modified plasma concentrations of several microbiota-derived metabolites. Most notably, metformin abolished the HFS-induced elevation of *p*-cresol sulfate, a tyrosine-derived uremic toxin linked to insulin resistance in muscle cells [56]. Metformin also partially rescued HFS-induced reduction in homoarginine, a protective metabolite against cardiometabolic risk [57]. However, metformin did not restore HFS-suppressed indole derivatives, despite a strong induction of *L. reuteri*, which is known to produce indoles with protective metabolic effects [58]. This suggests that indole signaling is not a primary pathway in metformin's beneficial action under our experimental conditions. Although SCFAs were not quantified in our study, previous reports showed that long-term metformin treatment increased circulating acetate, butyrate, and valerate levels in overweight adults, with acetate inversely correlating with fasting insulinemia [25].

This study aimed to dissect how metformin-induced changes in the intestinal tract contribute to improved glucose tolerance and reduced hyperinsulinemia in HFS-fed mice. Metformin impact on these metabolic parameters was clearly dose-dependent. Among the gut-related effects, only a subset, such as mucus production, epithelial proliferation (*Cyclin D1*), endocrine differentiation (*Ngn3*), GLP-1 expression, and shifts in some key bacterial taxa, showed a parallel dose–response pattern, supporting their involvement in metformin's metabolic improvement. In contrast, other changes, including ileal Fxr-Fgf15 inhibition and hepatic ceramide reduction, did not scale with dose, suggesting that they are not the main drivers of metformin dose-dependent effects on glycemic control. However, considering the importance of FXR and ceramides in insulin resistance, it is likely that their modulation contributes to the global antidiabetic action of metformin.

The study has some limitations. Because the different parameters were not measured in the same experiments and the same animals, and for some of them at only one metformin concentration, we did not perform correlation analyses between metformin doses, gut parameters, and glucose/insulin endpoints, hence limiting the demonstration of causal relationships. Also, despite an increase in the number of GLP-1-producing cells and stimulation of *Gcg* expression, the implication of GLP-1 cannot be firmly confirmed, since the circulating concentration of GLP-1 was not measured. Finally, only male mice were investigated, and thus it remains to determine whether sex differences exist in the mechanism of action of metformin in this model.

Despite these limitations, this study provides the first integrated analysis of metformin's dose-dependent effects on various intestinal functions, microbiota composition and host metabolism. The differential sensitivity of gut bacteria to metformin offers new insights into the drug's mechanism and suggests potential microbial biomarkers or targets for enhancing therapeutic efficacy. Further studies integrating dose and time-course analyses are warranted to disentangle early versus chronic effects of metformin, especially in light of recent evidence supporting its acute metabolic benefits in both preclinical [59] and clinical [60] contexts, and to fully understand how the gut-driven mechanisms shape metformin antidiabetic action.

## 4 Materials and methods

### 4.1 Animals, diet and metformin treatment

Five-week-old male C57BL/6J/Ola/Hsd mice (ENVIGO, Gannat, France) were housed in a temperature-controlled room (22 ± 2 °C) with a 12 h light/dark cycle. After one week of adaptation, animals were divided into six groups (n = 5 per group). The study was repeated at least three times. Control mice (SD) received standard chow (R16, GENOBIOS, Laval, France), while HFS groups were fed a high-fat high-sugar diet (260 HFF, SAFE, Augy, France; Supplementary Table 2) for 10 weeks. Metformin was then administered for 4 additional weeks via daily intragastric gavage, while maintaining HFS feeding. Metformin (PHR1084, Sigma, France) was delivered at 50, 100, or 200 mg/kg/day (MET50, MET100, MET200). Metformin was administrated at the end of the light phase, before the active feeding period. Control groups (SD and HFS) received water gavage. A separate experiment used 300 mg/kg/day of metformin (MET300) was also performed. All procedures followed ARRIVE guidelines and European legislation (directive 86/609/EEC), and the protocols were approved by the Rhône-Alpes Ethics Committee (CECCAPP, protocol LS-2023-002).

### 4.2 Body composition measurement

Lean, fat, and fluid mass were assessed using a Bruker Minispec Plus NMR analyzer (www.bruker.com), following the manufacturer's instructions.

### 4.3 Glucose tolerance test (ipGTT)

After 6 h fasting, mice received an intraperitoneal injection of d-glucose (1 g/kg). Blood glucose was measured at 15, 30, 45, 60, and 90 min post-injection. The glucose AUC was used to estimate glucose tolerance, as previously described [9].

### 4.4 Glucose and insulin assays

Plasma glucose and insulin levels were quantified using Glucose-Glo (Promega, France) and Ultrasensitive Insulin Assay (Eurobio Scientific, France). HOMA-IR index was calculated as [(fasting plasma glucose x Fasting serum Insulin)/22.5].

### 4.5 Blood and tissue sampling

After 6 h fasting, mice were euthanized by cervical dislocation. Blood was collected, and ileum and colon were harvested, divided, and processed either for RNA (flushed with PBS and frozen in liquid nitrogen) or for microbiota analysis (frozen unflushed). Caecum and liver were collected and snap-frozen. Portions of liver, ileum, and colon were fixed in 4% paraformaldehyde and paraffin-embedded.

### 4.6 Triglyceride assay

Lipids were extracted from 10 mg liver powder using the Lipid Extraction Kit (ab211044, Abcam). Triglycerides were measured with Biolabo reagent (87,319), following the manufacturer's instructions [30].

### 4.7 Gene expression analyses

Total RNA was extracted using TRI Reagent (T9424, Sigma). cDNA was analyzed by RT-qPCR (Rotor-Gene, QIAGEN). Data were normalized to *Tbp* mRNA as previously reported [9,30]. A standard curve was included in all assays. PCR primers are listed in Supplementary Table 3.

### 4.8 Bile acid profiling

Bile acids were quantified in 100 mg frozen caecum by HPLC-MS/MS according to Humbert et al. [61] as previously reported [9,30]. Data are expressed in nmol/g of wet tissue.

### 4.9 Microbiota analysis

Total DNA was extracted from different intestinal sections using the ZymoBIOMICS DNA Microprep Kit, (D4300T, Zymo Research, France). Library preparation and sequencing were outsourced to the GenEPII platform (https://teamhcl.chu-lyon.fr/genepii), with the Quick-16S Plus NGS Library Prep Kit (V3–V4) (Zymo research). Sequencing was performed on Illumina MiSeq using a paired-end 2∗300 strategy. Quality of sequenced reads was determined using FastQC and sequences were then analyzed using an in-house QIIME2 based pipeline (trimming, denoising and chimera filtering using the DADA2 plugin, ASV annotation and taxonomic classification using scikit-learn). Raw counts were then rarefied, Alpha and Beta diversities were calculated under RStudio (2024.09.0 Build 375) using the phyloseq (v1.48.0) library. MaAsLin2 (1.18.0) was used to identified differentially abundant ASV. FASTQ data deposited in the French repository at “Recherche.data.gouv.fr” accessible with the following link: https://entrepot.recherche.data.gouv.fr/privateurl.xhtml?token=46ced6c9-7ddf-405a-a510-7bc45f77069d.

### 4.10 Lipid droplet and mucus staining

Liver sections (5 μm) were deparaffinized in xylene and rehydrated through a graded ethanol series. Then, section were stained with Hematoxylin/Phloxin for nuclear/cytoplasmic contrast; lipid droplets appeared as white voids. For mucus detection in colon, Alcian blue (B8438, Sigma Aldrich) staining was performed. Images were acquired using Microscope Olympus BX63 (Olympus, France) and analyzed with the CellSens Dimension software (Olympus). Mucus area was quantified using ImageJ and expressed as percentage of analyzed surface (villi + crypts).

### **4.11** Immunohistochemistry

Deparaffinized sections were rehydrated, and antigen-retrieved. Slides were incubated for 2 h at room temperature with anti-GLP1 (ab23472) or anti-Ki67 (ab16667) (1:50; Abcam, France). Secondary antibodies (AF594 goat anti-mouse, A11005; AF594 goat anti-rat IgG, A11012; 1:500, Invitrogen) were added for 1 h. Slides were mounted with Mounting Medium with DAPI (ab104139, Abcam). Images were captured using Microscope Olympus BX63 and analyzed with the CellSens Dimension software (Olympus).

### **4.12** Ceramide profiling

Liver ceramides were extracted with 2.5 mL chloroform/methanol (1:2, v/v) containing internal standard (C17:0-ceramide, Avanti Polar Lipids). After 2 h shaking and centrifugation (1900 g, 10 min, room temp.), samples were dried under nitrogen, resuspended in 1 mL chloroform/methanol, and analyzed via direct flow injection on a triple quadrupole MS (API 4500 QTRAP; Sciex) in MRM mode [62]. Total ceramides were expressed as nmol/g liver protein.

### 4.13 Metabolomics analysis

Metabolomics was performed at the Metanutribiota platform (CarMeN lab, Hôpital Lyon-Sud). Plasma (10 μL) was analyzed using the MxP Quant 500 kit (Biocrates Life Sciences) on a XEVO TQ-XS® UPLC-MS system. Sample preparation, QC, and quantification followed manufacturer instructions. Data were processed with MassLynx® and WebIDQ™. Metabolites with >20% missing values across all groups were excluded. Statistical analysis was done in MetaboAnalyst 6.0. Data were autoscaled; PLS-DA identified discriminant metabolites based on VIP scores.

### 4.14 Statistical analyses

Data are presented as mean ± SEM. One-way ANOVA followed by Tukey post-hoc test was used to compare Metformin-treated groups to HFS controls (GraphPad Prism 9, GraphPad Software). Significance: ∗p < 0.05, ∗∗p < 0.01, ∗∗∗p < 0.001; $p < 0.05, #p < 0.01, ¤p < 0.001 (Tukey test).

## CRediT authorship contribution statement

**Murielle Godet:** Writing – review & editing, Writing – original draft, Conceptualization, Investigation, Validation, Data curation, Formal analysis. **Emmanuelle Meugnier:** Writing – review & editing, Validation, Formal analysis, Data curation. **Oriane Vitalis:** Writing – review & editing, Investigation. **Nadia Bendridi:** Writing – review & editing, Investigation. **Aurélie Vieille-Marchiset:** Writing – review & editing, Investigation. **Nathalie Vega:** Writing – review & editing, Investigation. **Bérengère Benoit:** Writing – review & editing, Writing – original draft. **Claudie Pinteur:** Writing – review & editing, Investigation. **Dominique Rainteau:** Writing – review & editing, Validation, Investigation, Data curation. **David Cheillan:** Writing – review & editing, Validation, Investigation, Data curation. **Marie-Caroline Michalski:** Writing – review & editing, Resources, Validation. **Karim Chikh:** Writing – review & editing, Investigation, Data curation. **Hubert Vidal:** Writing – review & editing, Writing – original draft, Validation, Supervision, Resources, Project administration, Funding acquisition, Conceptualization.

## Declaration of Generative AI and AI-assisted technologies in the Writing process

During the preparation of this manuscript, the authors used ChatGPT in order to reduce the number of words of the main text. After using this tool, the authors reviewed and edited the content as needed and take full responsibility for the content of the published article.

## Funding

This work was funded by grants from the “Fondation Francophone pour la Recherche sur le Diabète” and from INSERM Transversal Program on Microbiota (PTM).

## Declaration of competing interest

The authors declare no conflicts of interest.

## Data Availability

Data will be made available on request.
