# Supplementary material for: Evaluation of the effects of metformin on gut functions and microbiota and their contribution to improving glucose tolerance in diabetic mice
Source: Mol Metab. 2025 Sep 29;102:102263. doi: 10.1016/j.molmet.2025.102263 (PMC12547773; doi:10.1016/j.molmet.2025.102263)
Supplement: Multimedia component 1 [file mmc1.pptx]

## Slide 1
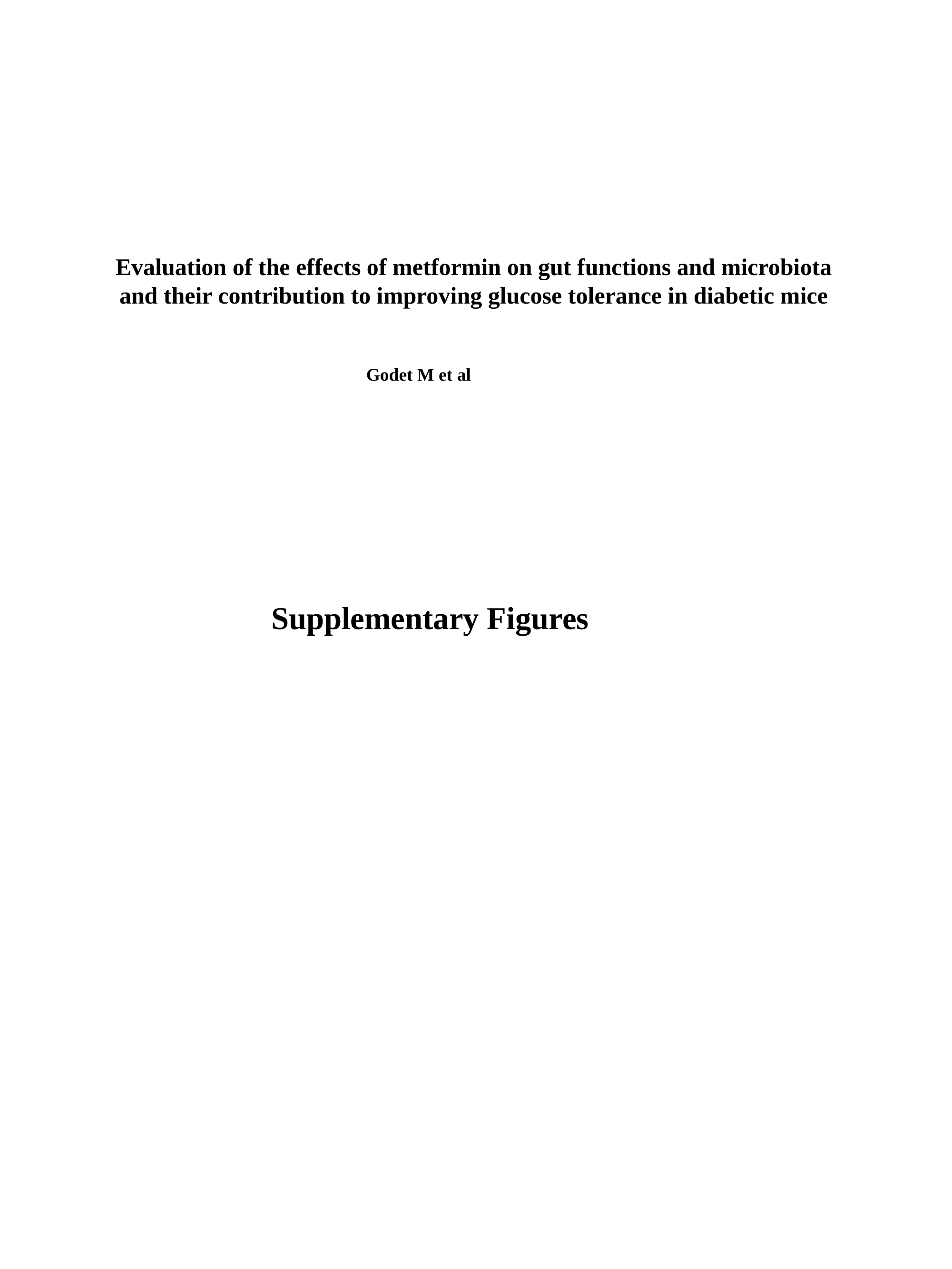

Evaluation of the effects of metformin on gut functions and microbiota and their contribution to improving glucose tolerance in diabetic mice
Godet M et al
Supplementary Figures

## Slide 2
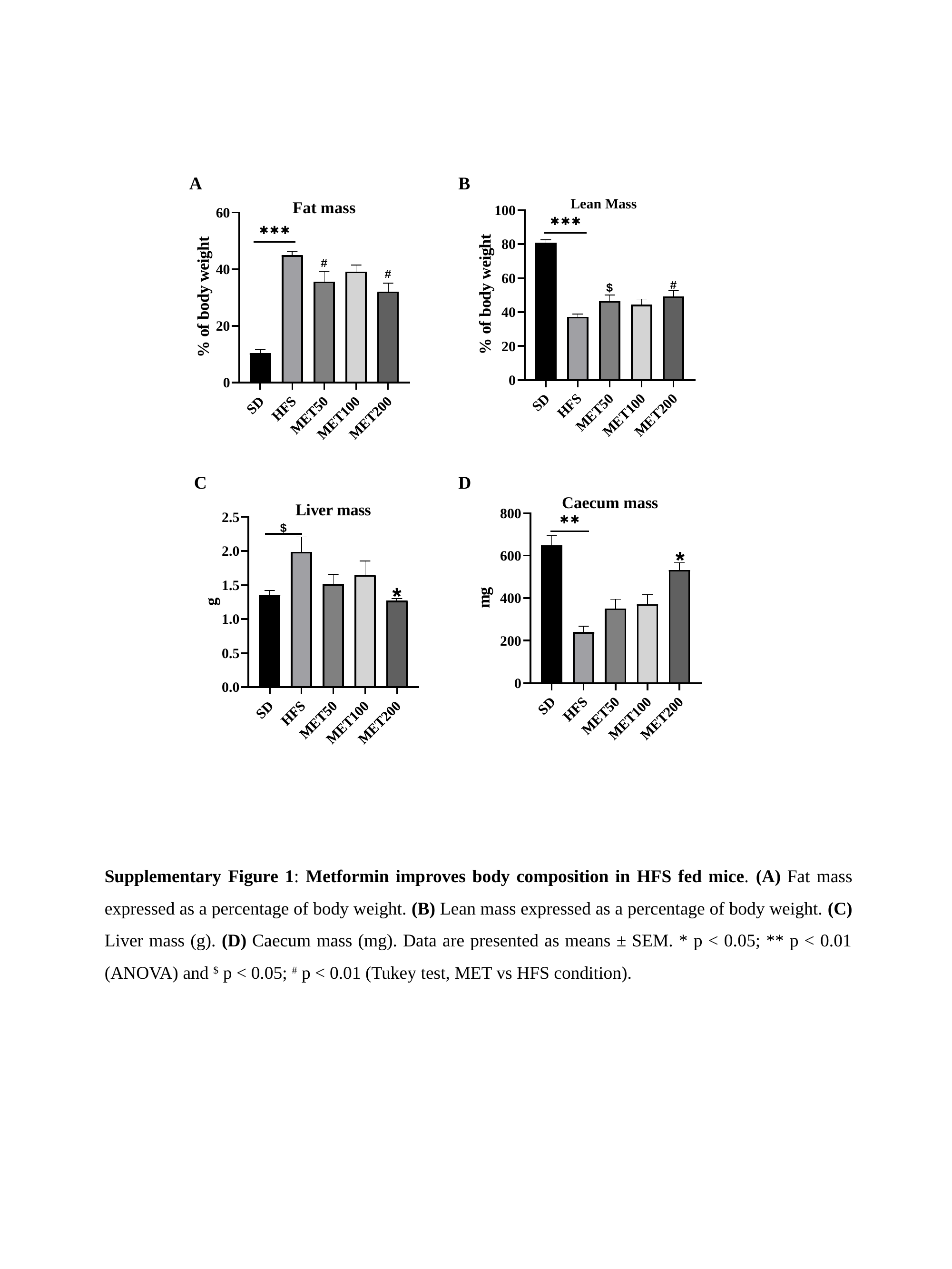

A
B
C
D
Supplementary Figure 1: Metformin improves body composition in HFS fed mice. (A) Fat mass expressed as a percentage of body weight. (B) Lean mass expressed as a percentage of body weight. (C) Liver mass (g). (D) Caecum mass (mg). Data are presented as means ± SEM. * p < 0.05; ** p < 0.01 (ANOVA) and $ p < 0.05; # p < 0.01 (Tukey test, MET vs HFS condition).

## Slide 3
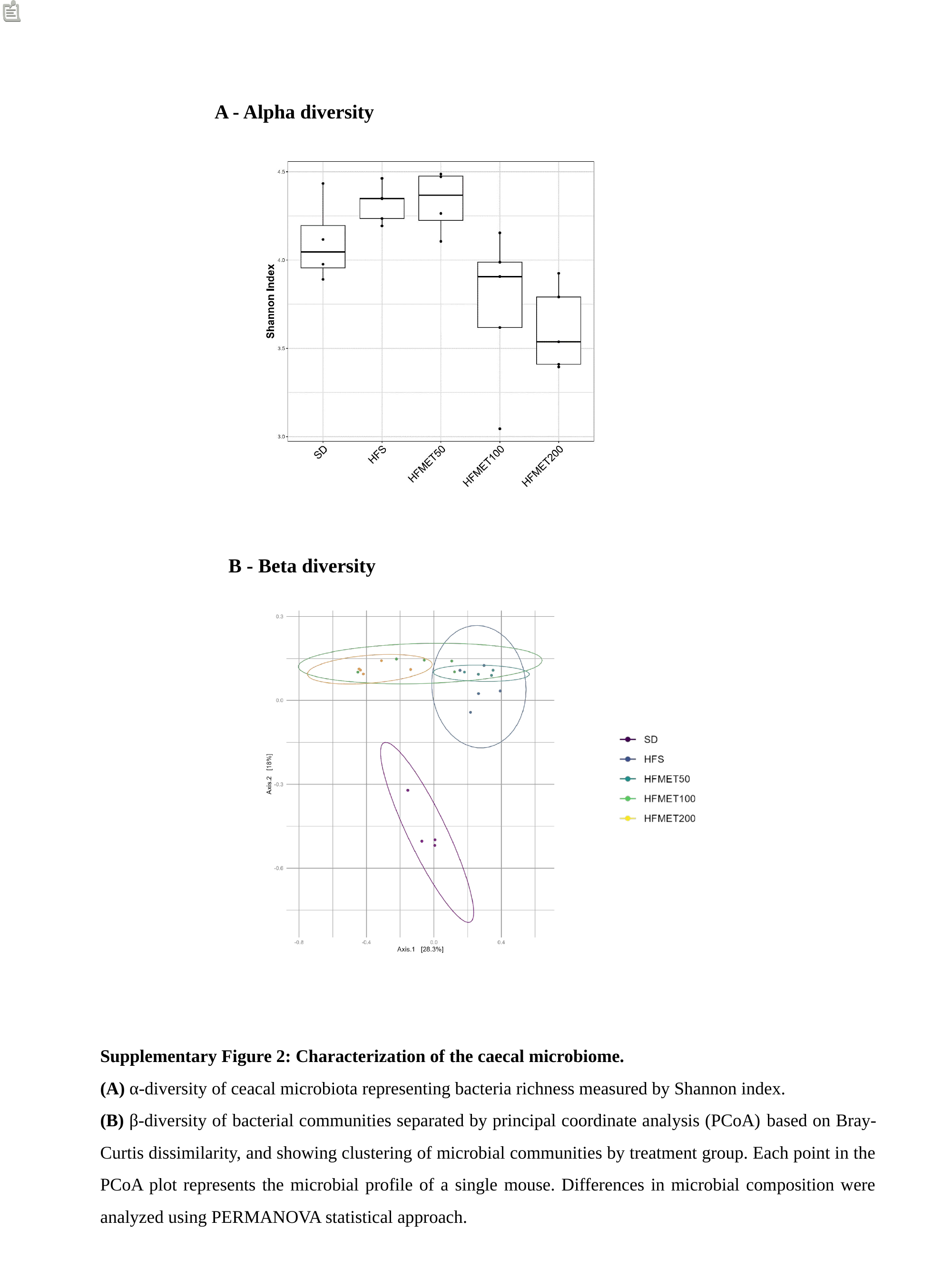

A - Alpha diversity
B - Beta diversity
Supplementary Figure 2: Characterization of the caecal microbiome.
(A) α-diversity of ceacal microbiota representing bacteria richness measured by Shannon index.
(B) β-diversity of bacterial communities separated by principal coordinate analysis (PCoA) based on Bray-Curtis dissimilarity, and showing clustering of microbial communities by treatment group. Each point in the PCoA plot represents the microbial profile of a single mouse. Differences in microbial composition were analyzed using PERMANOVA statistical approach.

## Slide 4
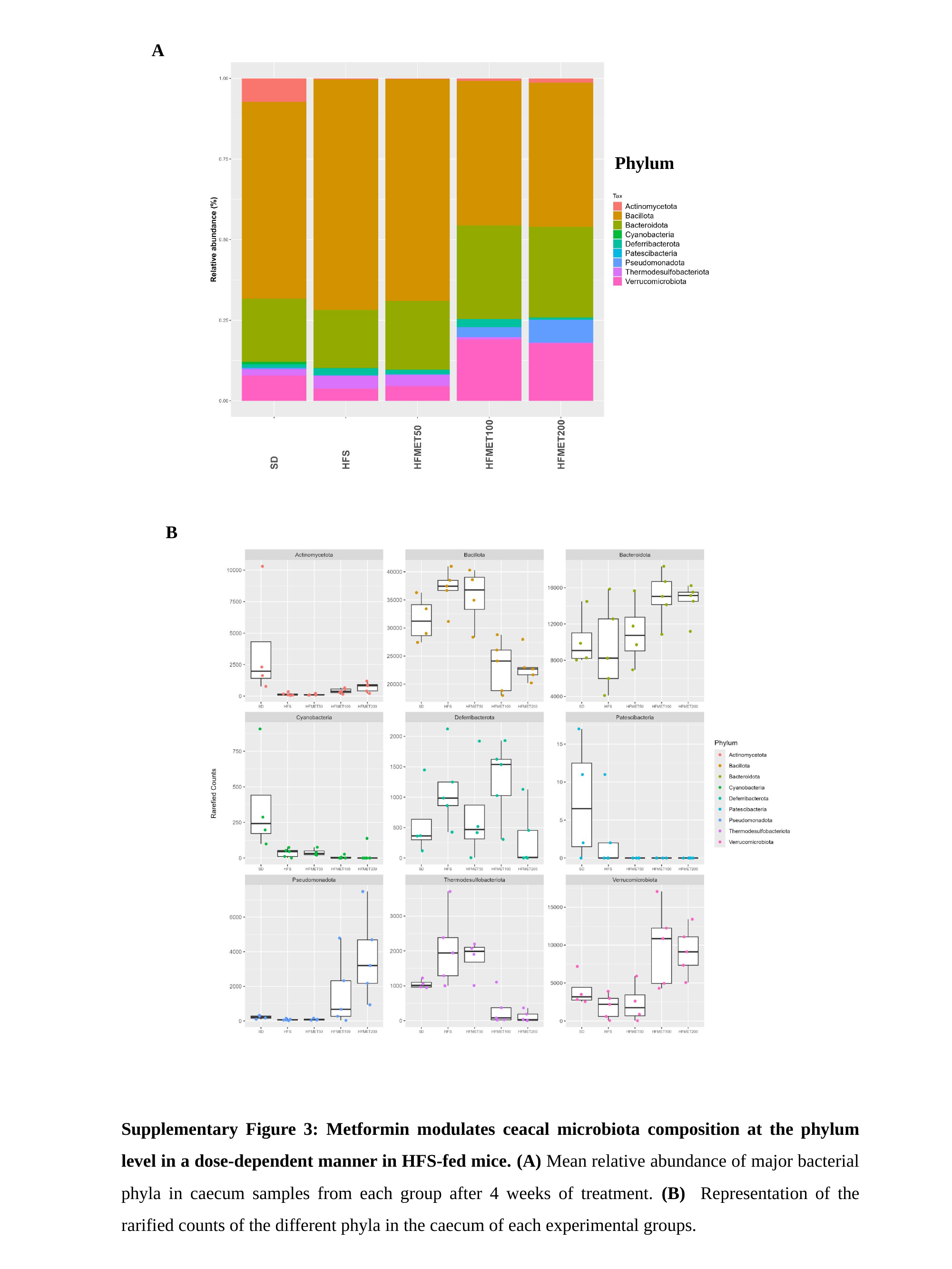

A
Phylum
B
Supplementary Figure 3: Metformin modulates ceacal microbiota composition at the phylum level in a dose-dependent manner in HFS-fed mice. (A) Mean relative abundance of major bacterial phyla in caecum samples from each group after 4 weeks of treatment. (B) Representation of the rarified counts of the different phyla in the caecum of each experimental groups.

## Slide 5
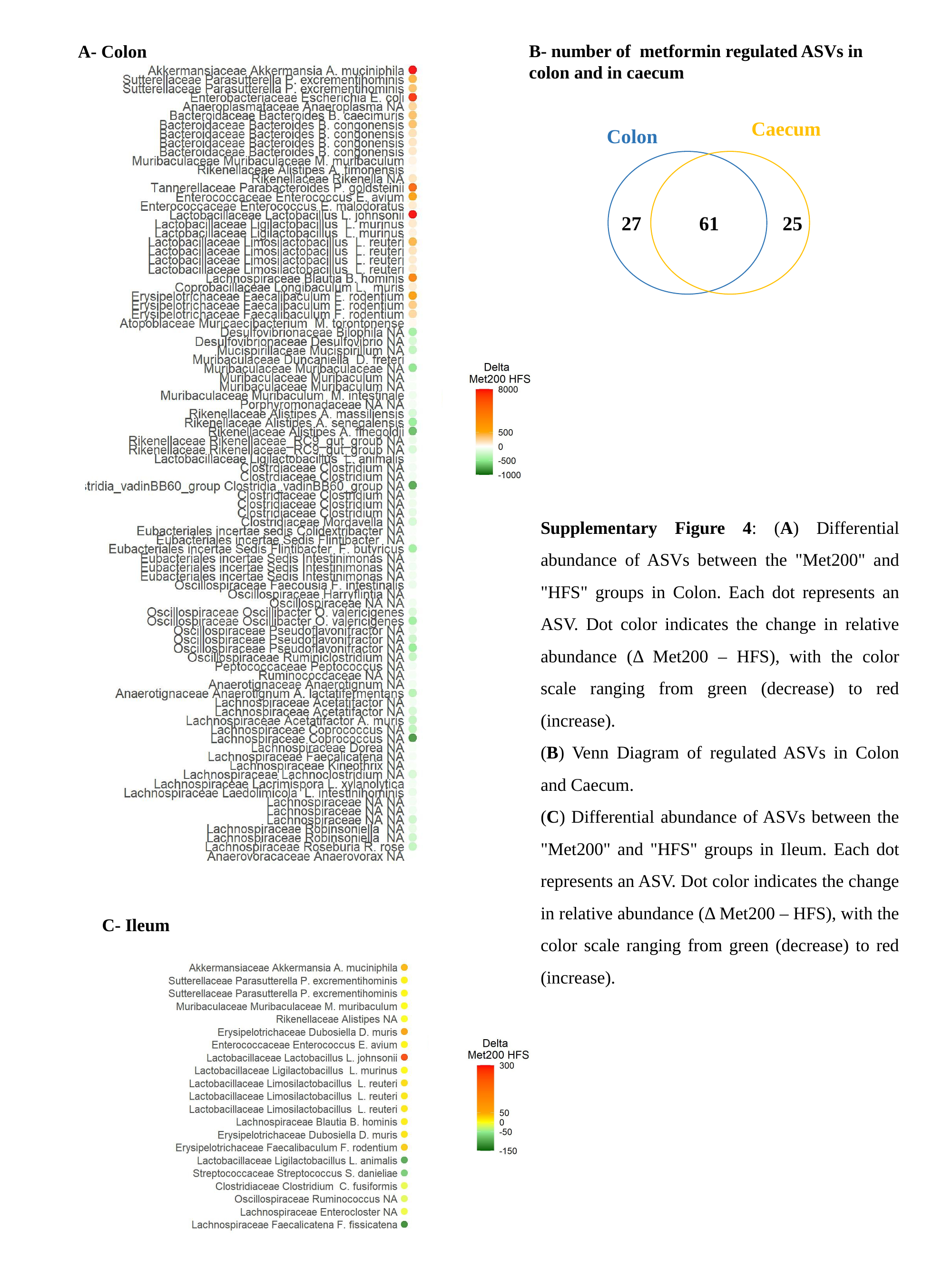

B- number of metformin regulated ASVs in colon and in caecum
A- Colon
Caecum
Colon
27
61
25
Supplementary Figure 4: (A) Differential abundance of ASVs between the "Met200" and "HFS" groups in Colon. Each dot represents an ASV. Dot color indicates the change in relative abundance (Δ Met200 – HFS), with the color scale ranging from green (decrease) to red (increase).
(B) Venn Diagram of regulated ASVs in Colon and Caecum.
(C) Differential abundance of ASVs between the "Met200" and "HFS" groups in Ileum. Each dot represents an ASV. Dot color indicates the change in relative abundance (Δ Met200 – HFS), with the color scale ranging from green (decrease) to red (increase).
C- Ileum

## Slide 6
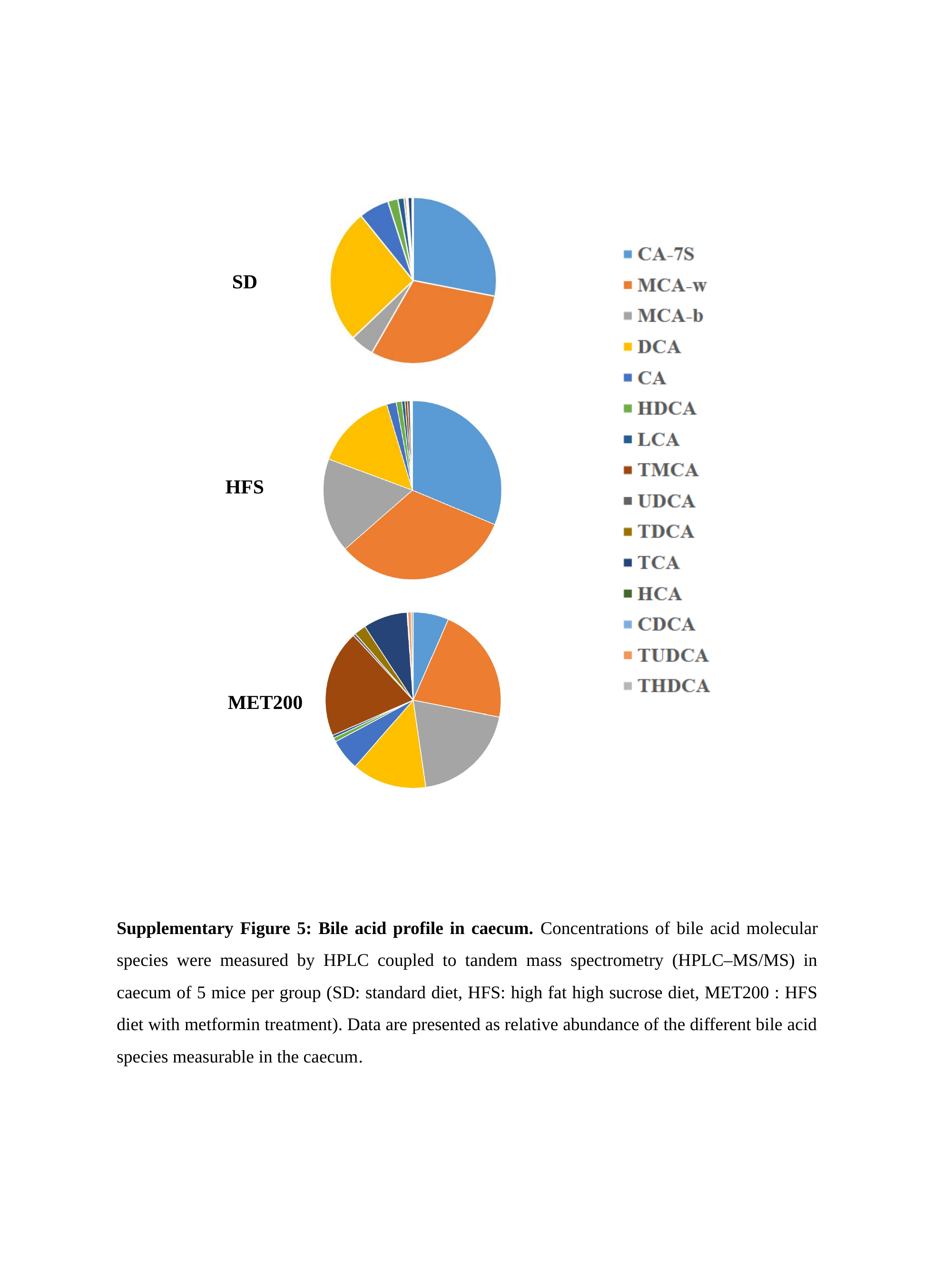

SD
HFS
MET200
Supplementary Figure 5: Bile acid profile in caecum. Concentrations of bile acid molecular species were measured by HPLC coupled to tandem mass spectrometry (HPLC–MS/MS) in caecum of 5 mice per group (SD: standard diet, HFS: high fat high sucrose diet, MET200 : HFS diet with metformin treatment). Data are presented as relative abundance of the different bile acid species measurable in the caecum.

## Slide 7
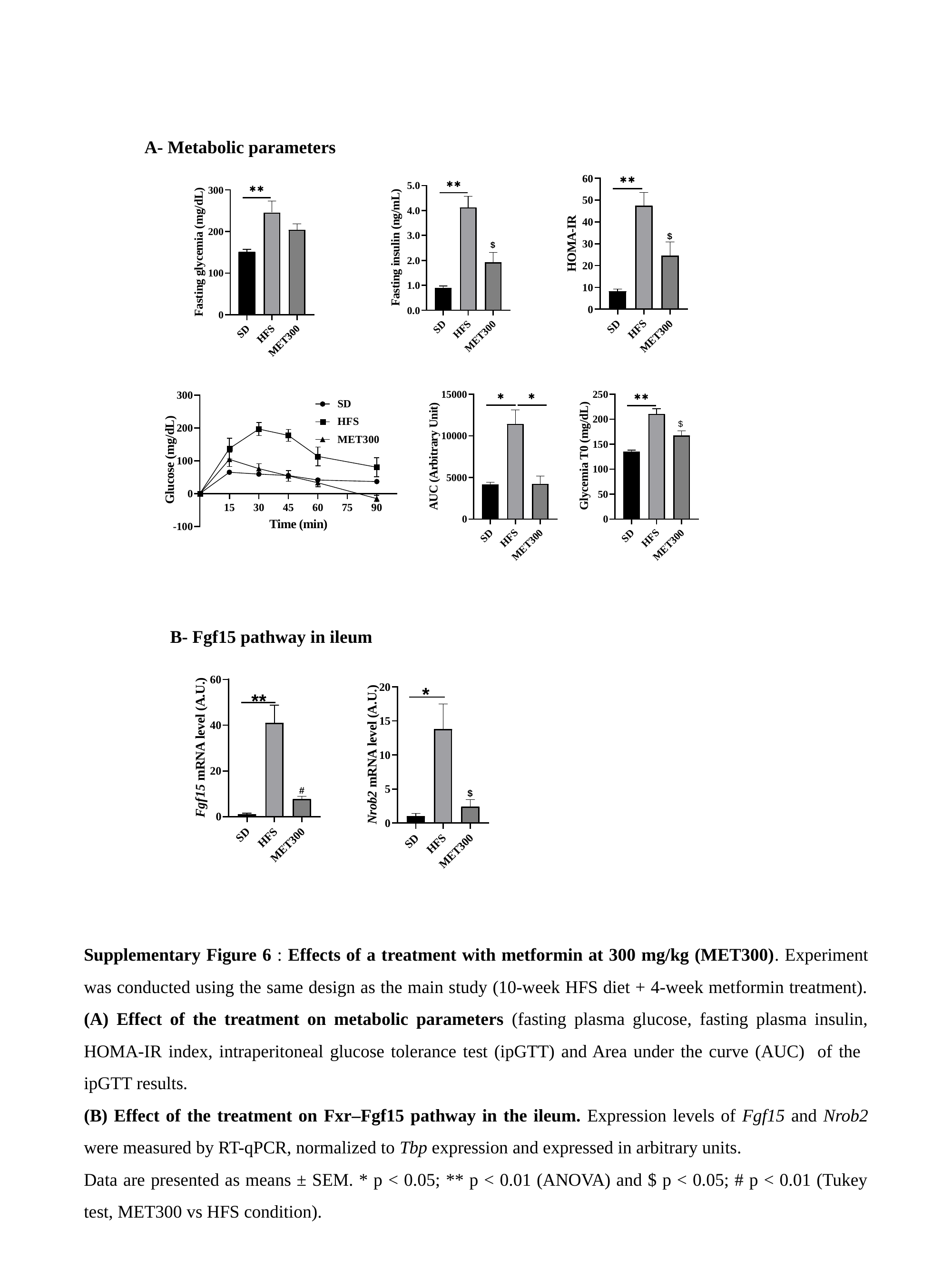

A- Metabolic parameters
B- Fgf15 pathway in ileum
Supplementary Figure 6 : Effects of a treatment with metformin at 300 mg/kg (MET300). Experiment was conducted using the same design as the main study (10-week HFS diet + 4-week metformin treatment). (A) Effect of the treatment on metabolic parameters (fasting plasma glucose, fasting plasma insulin, HOMA-IR index, intraperitoneal glucose tolerance test (ipGTT) and Area under the curve (AUC) of the ipGTT results.
(B) Effect of the treatment on Fxr–Fgf15 pathway in the ileum. Expression levels of Fgf15 and Nrob2 were measured by RT-qPCR, normalized to Tbp expression and expressed in arbitrary units.
Data are presented as means ± SEM. * p < 0.05; ** p < 0.01 (ANOVA) and $ p < 0.05; # p < 0.01 (Tukey test, MET300 vs HFS condition).

## Slide 8
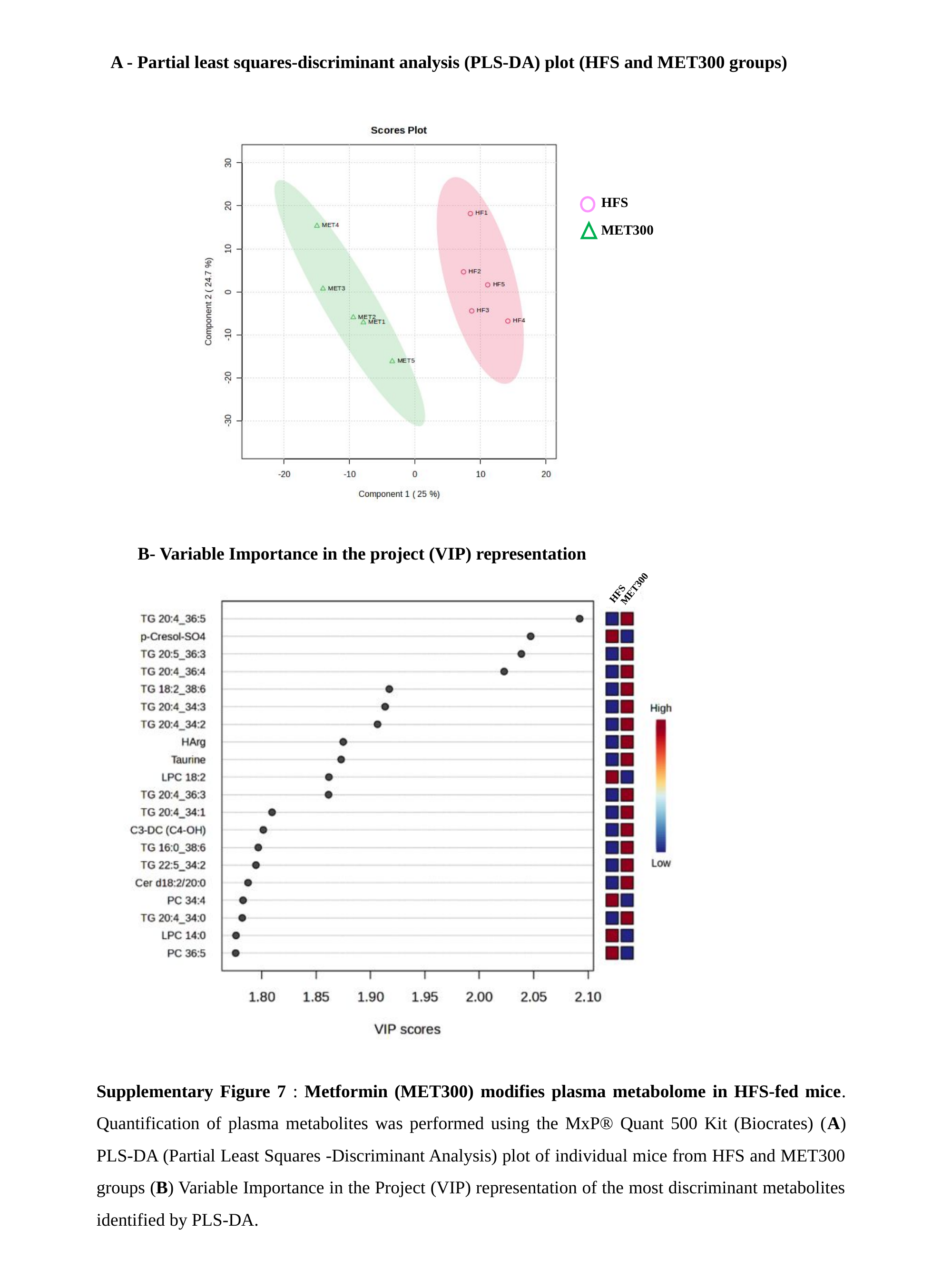

A - Partial least squares-discriminant analysis (PLS-DA) plot (HFS and MET300 groups)
HFS
MET300
MET300
HFS
B- Variable Importance in the project (VIP) representation
Supplementary Figure 7 : Metformin (MET300) modifies plasma metabolome in HFS-fed mice. Quantification of plasma metabolites was performed using the MxP® Quant 500 Kit (Biocrates) (A) PLS-DA (Partial Least Squares -Discriminant Analysis) plot of individual mice from HFS and MET300 groups (B) Variable Importance in the Project (VIP) representation of the most discriminant metabolites identified by PLS-DA.

## Slide 9
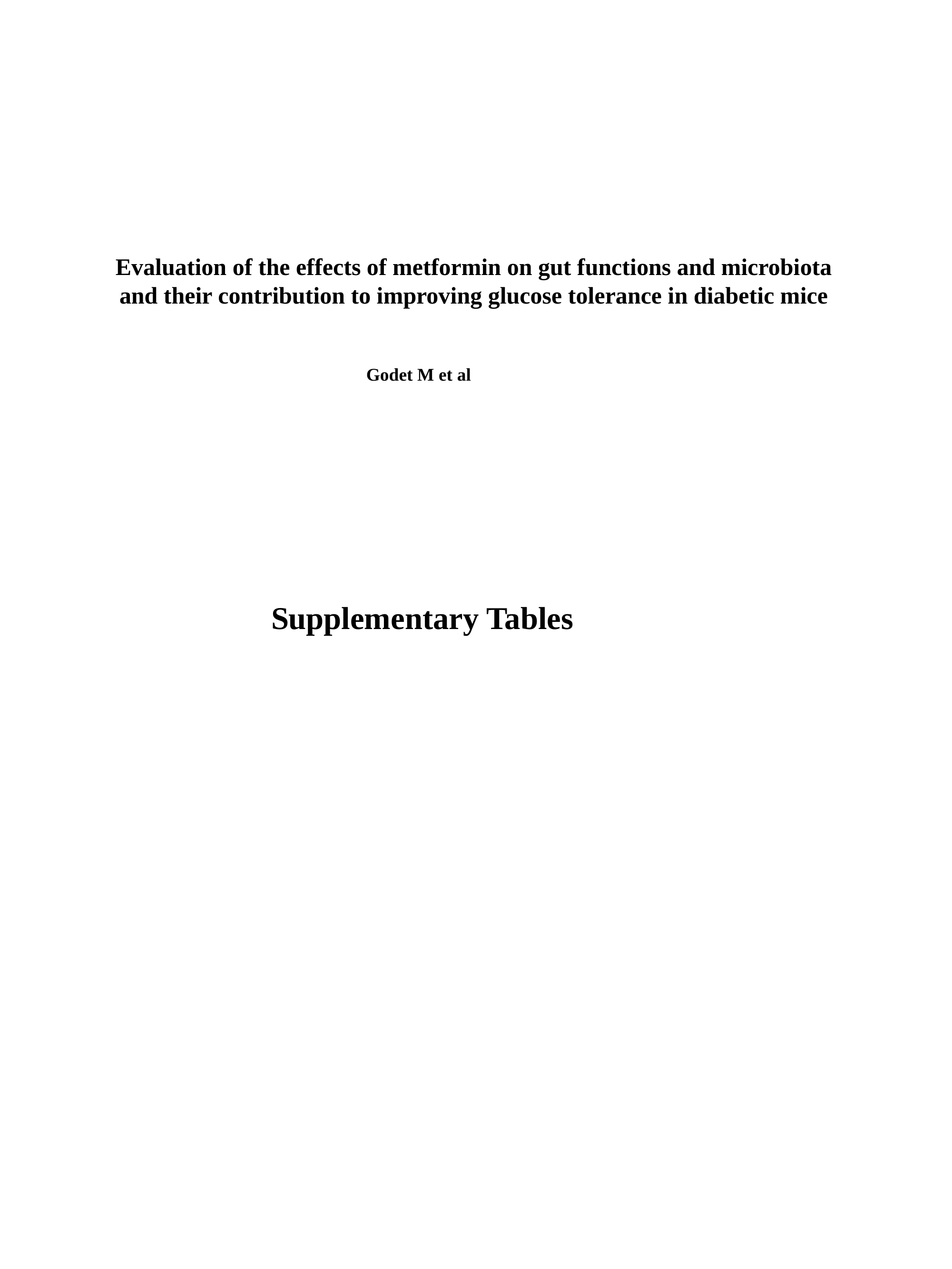

Evaluation of the effects of metformin on gut functions and microbiota and their contribution to improving glucose tolerance in diabetic mice
Godet M et al
Supplementary Tables

## Slide 10
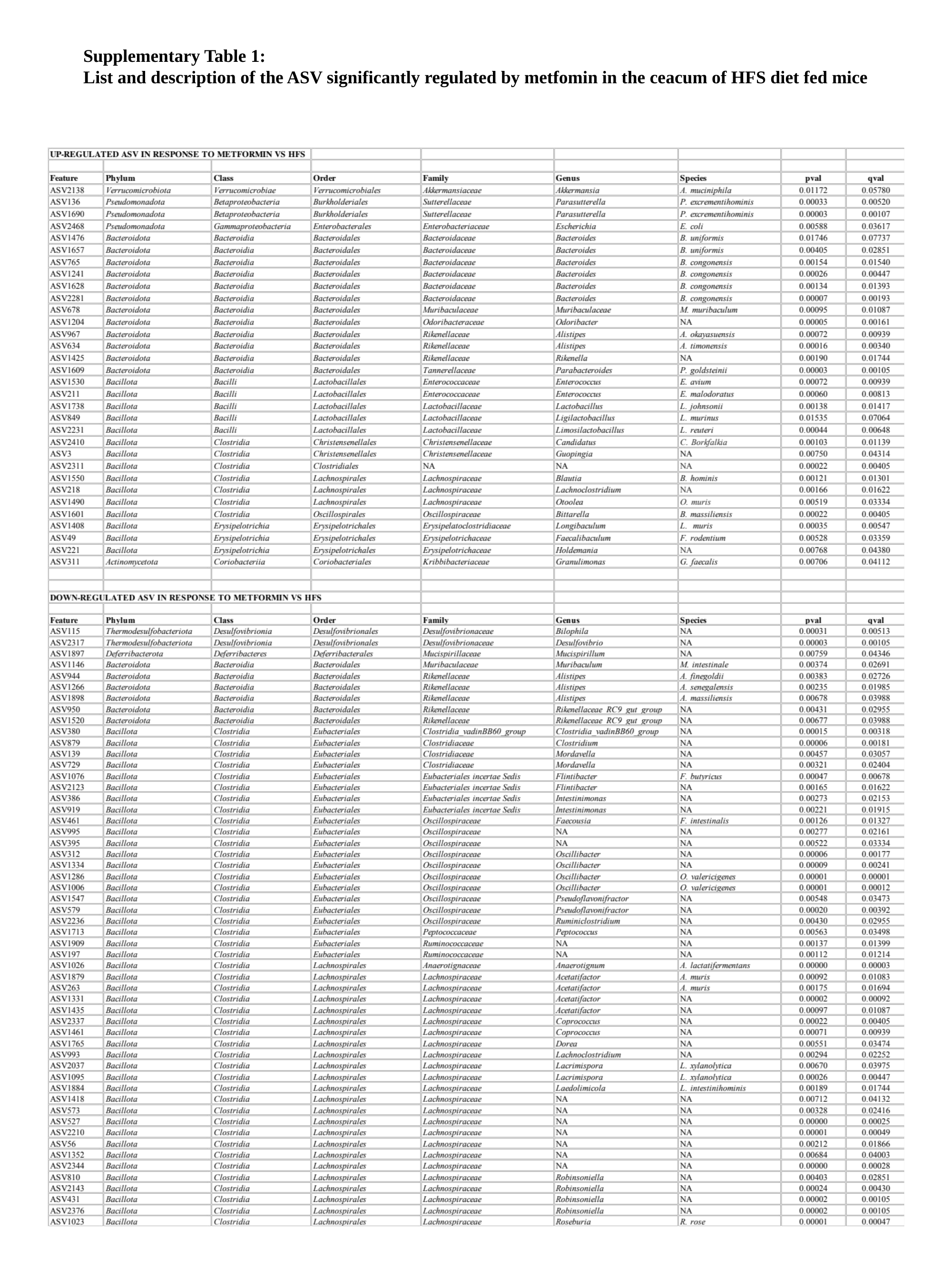

Supplementary Table 1:
List and description of the ASV significantly regulated by metfomin in the ceacum of HFS diet fed mice

## Slide 11
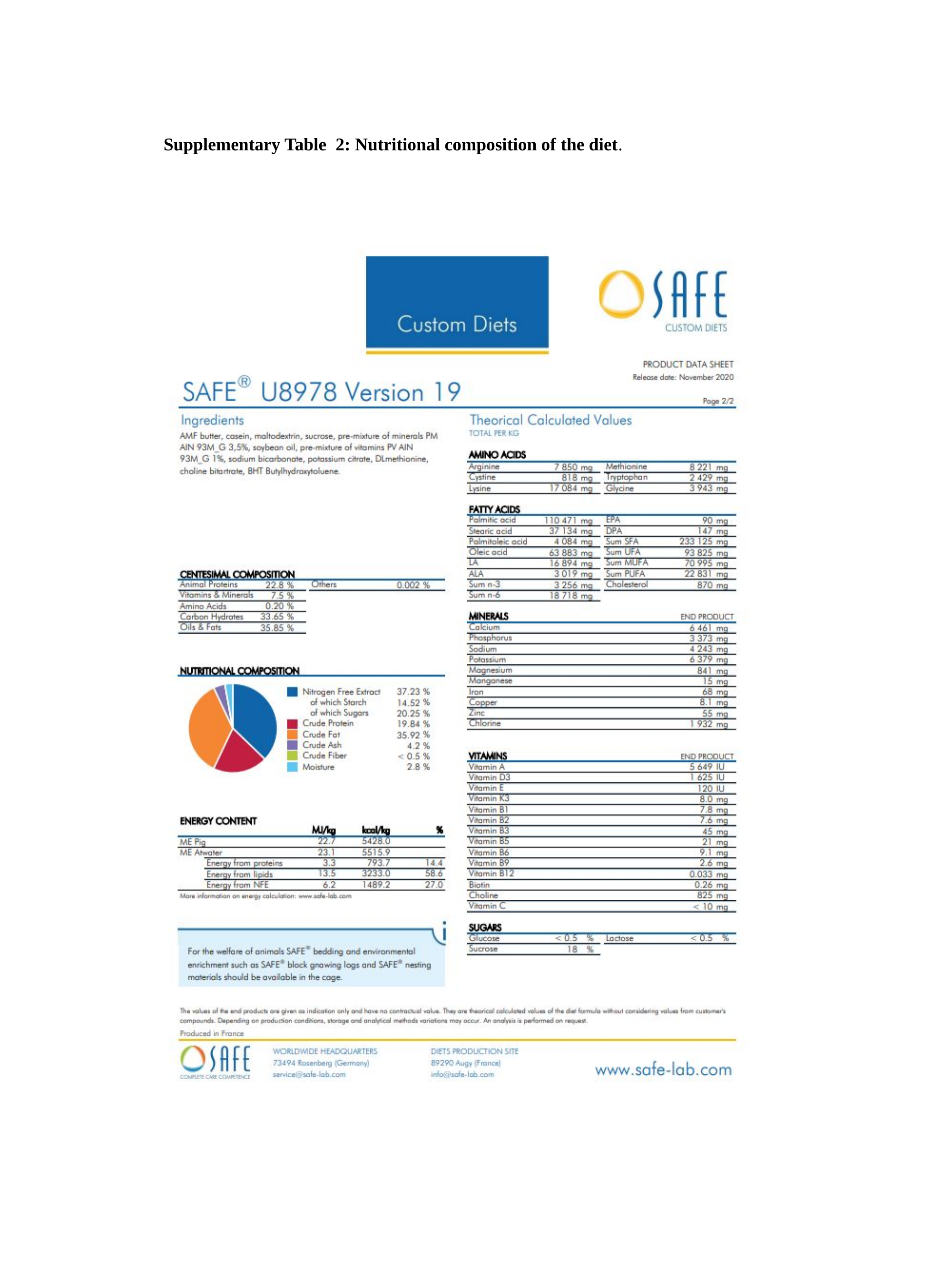

Supplementary Table 2: Nutritional composition of the diet.

## Slide 12
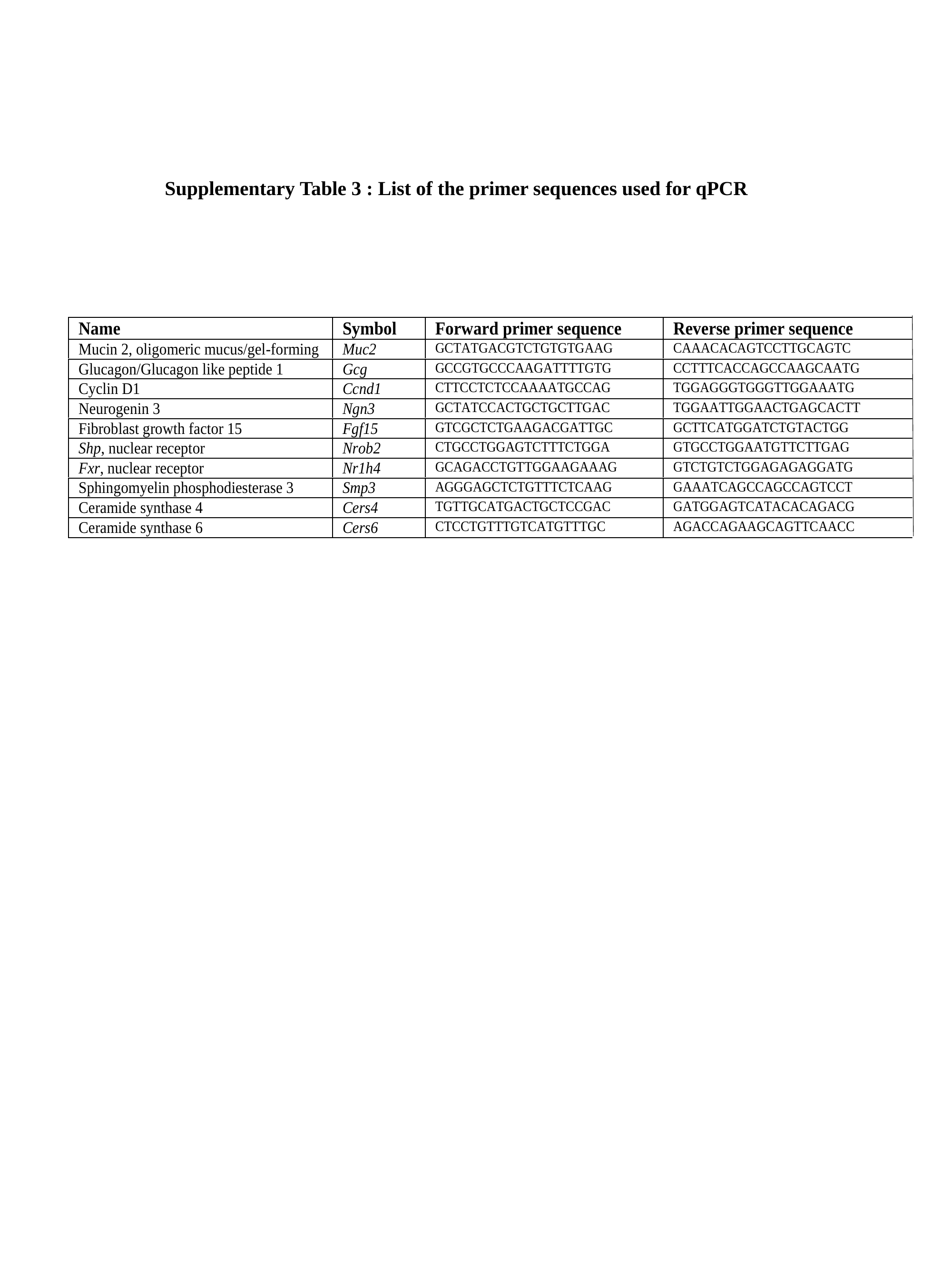

Supplementary Table 3 : List of the primer sequences used for qPCR
